# Respiratory viral infections in otherwise healthy humans with inherited IRF7 deficiency

**DOI:** 10.1084/jem.20220202

**Published:** 2022-06-07

**Authors:** Tessa Mollie Campbell, Zhiyong Liu, Qian Zhang, Marcela Moncada-Velez, Laura E. Covill, Peng Zhang, Ilad Alavi Darazam, Paul Bastard, Lucy Bizien, Giorgia Bucciol, Sara Lind Enoksson, Emmanuelle Jouanguy, Şemsi Nur Karabela, Taushif Khan, Yasemin Kendir-Demirkol, Andres Augusto Arias, Davood Mansouri, Per Marits, Nico Marr, Isabelle Migeotte, Leen Moens, Tayfun Ozcelik, Isabelle Pellier, Anton Sendel, Sevtap Şenoğlu, Mohammad Shahrooei, C.I. Edvard Smith, Isabelle Vandernoot, Karen Willekens, Kadriye Kart Yaşar, COVID Human Genetic Effort, Laurent Abel, Alessandro Aiuti, Saleh Al-Muhsen, Fahd Al-Mulla, Mark S. Anderson, Evangelos Andreakos, Andrés A. Arias, Hagit Baris Feldman, Alexandre Belot, Catherine M. Biggs, Dusan Bogunovic, Alexandre Bolze, Anastasiia Bondarenko, Ahmed A. Bousfiha, Petter Brodin, Yenan Bryceson, Carlos D. Bustamante, Manish J. Butte, Giorgio Casari, John Christodoulou, Antonio Condino-Neto, Stefan N. Constantinescu, Megan A. Cooper, Clifton L. Dalgard, Murkesh Desai, Beth A. Drolet, Jamila El Baghdadi, Sara Espinosa-Padilla, Jacques Fellay, Carlos Flores, José Luis Franco, Antoine Froidure, Peter K. Gregersen, Bodo Grimbacher, Filomeen Haerynck, David Hagin, Rabih Halwani, Lennart Hammarström, James R. Heath, Sarah E. Henrickson, Elena W.Y. Hsieh, Eystein Husebye, Kohsuke Imai, Yuval Itan, Erich D. Jarvis, Timokratis Karamitros, Kai Kisand, Cheng-Lung Ku, Yu-Lung Lau, Yun Ling, Carrie L. Lucas, Tom Maniatis, Davood Mansouri, László Maródi, Isabelle Meyts, Joshua D. Milner, Kristina Mironska, Trine H. Mogensen, Tomohiro Morio, Lisa F.P. Ng, Luigi D. Notarangelo, Antonio Novelli, Giuseppe Novelli, Cliona O'Farrelly, Satoshi Okada, Keisuke Okamoto, Tayfun Ozcelik, Qiang Pan-Hammarström, Maria Papadaki, Jean W. Pape, Rebeca Perez de Diego, David S. Perlin, Graziano Pesole, Anna M. Planas, Carolina Prando, Aurora Pujol, Lluis Quintana-Murci, Sathishkumar Ramaswamy, Laurent Renia, Igor Resnick, Carlos Rodríguez-Gallego, Vanessa Sancho-Shimizu, Anna Sediva, Mikko R.J. Seppänen, Mohammed Shahrooei, Anna Shcherbina, Ondrej Slaby, Andrew L. Snow, Pere Soler-Palacín, András N. Spaan, Ivan Tancevski, Stuart G. Tangye, Ahmad Abou Tayoun, Stuart E. Turvey, K M Furkan Uddin, Mohammed J. Uddin, Diederik van de Beek, Donald C. Vinh, Horst von Bernuth, Joost Wauters, Mayana Zatz, Pawel Zawadzki, Helen C. Su, Jean-Laurent Casanova, Peter Bergman, Laurent Abel, Aurélie Cobat, Jean-Laurent Casanova, Isabelle Meyts, Yenan T. Bryceson

**Affiliations:** 1 Center for Hematology and Regenerative Medicine, Department of Medicine, Karolinska Institutet, Stockholm, Sweden; 2 St. Giles Laboratory of Human Genetics of Infectious Diseases, Rockefeller Branch, Rockefeller University, New York, NY; 3 Laboratory of Human Genetics of Infectious Diseases, Necker Branch, INSERM U1163, Necker Hospital for Sick Children, Paris, France; 4 University Paris Cité, Imagine Institute, Paris, France; 5 Department of Infectious Diseases and Tropical Medicine, Loghman Hakim Hospital, Shahid Beheshti University of Medical Sciences, Tehran, Iran; 6 Infectious Diseases and Tropical Medicine Research Center, Shahid Beheshti University of Medical Sciences, Tehran, Iran; 7 Department of Microbiology, Immunology and Transplantation, Laboratory of Inborn Errors of Immunity, KU Leuven, Leuven, Belgium; 8 Department of Pediatrics, University Hospitals Leuven, Leuven, Belgium; 9 Department of Clinical Immunology and Transfusion Medicine, Karolinska University Hospital, Stockholm, Sweden; 10 Department of Clinical Science, Intervention and Technology, Karolinska Institutet, Stockholm, Sweden; 11 Department of Infectious Diseases and Clinical Microbiology, Bakirkoy Dr. Sadi Konuk Training and Research Hospital, University of Health Sciences, Istanbul, Turkey; 12 Department of Human Immunology, Research Branch, Sidra Medicine, Doha, Qatar; 13 Primary Immunodeficiencies Group, University of Antioquia UdeA, Medellin, Colombia; 14 School of Microbiology, University of Antioquia UdeA, Medellin, Colombia; 15 Department of Clinical Immunology and Infectious Diseases, National Research Institute of Tuberculosis and Lung Diseases, Shahid Beheshti University of Medical Sciences, Tehran, Iran; 16 The Clinical Tuberculosis and Epidemiology Research Center, National Research Institute of Tuberculosis and Lung Diseases, Masih Daneshvari Hospital, Shahid Beheshti University of Medical Sciences, Tehran, Iran; 17 Centre de Génétique Humaine de l’Université Libre de Bruxelles, Hôpital Erasme, Brussels, Belgium; 18 Department of Molecular Biology and Genetics, Bilkent University, Bilkent-Ankara, Turkey; 19 Université d'Angers, INSERM, CNRS, CRCI^2^NA, Pediatric Immuno-Hemato-oncology Unit, CHU Angers, Angers, France; 20 Specialized Immunology Laboratory of Dr. Shahrooei, Sina Medical Complex, Ahvaz, Iran; 21 Department of Microbiology and Immunology, Clinical and Diagnostic Immunology, KU Leuven, Leuven, Belgium; 22 Department of Infectious Diseases, The Immunodeficiency Unit, Karolinska University Hospital, Stockholm, Sweden; 23 Department of Laboratory Medicine, Translational Research Center Karolinska, Karolinska Institutet, Stockholm, Sweden; 24 Department of Molecular Genetics, University Hospitals Leuven, Leuven, Belgium; 25 Department of Laboratory Medicine, Division of Clinical Microbiology, Karolinska Institutet, Stockholm, Sweden; 26 Howard Hughes Medical Institute, New York, NY; 27 Department of Pediatrics, Necker Hospital for Sick Children, Paris, France; 28 Broegelmann Laboratory, Department of Clinical Sciences, University of Bergen, Bergen, Norway

## Abstract

Autosomal recessive IRF7 deficiency was previously reported in three patients with single critical influenza or COVID-19 pneumonia episodes. The patients’ fibroblasts and plasmacytoid dendritic cells produced no detectable type I and III IFNs, except IFN-β. Having discovered four new patients, we describe the genetic, immunological, and clinical features of seven IRF7-deficient patients from six families and five ancestries. Five were homozygous and two were compound heterozygous for IRF7 variants. Patients typically had one episode of pulmonary viral disease. Age at onset was surprisingly broad, from 6 mo to 50 yr (mean age 29 yr). The respiratory viruses implicated included SARS-CoV-2, influenza virus, respiratory syncytial virus, and adenovirus. Serological analyses indicated previous infections with many common viruses. Cellular analyses revealed strong antiviral immunity and expanded populations of influenza- and SARS-CoV-2–specific memory CD4^+^ and CD8^+^ T cells. IRF7-deficient individuals are prone to viral infections of the respiratory tract but are otherwise healthy, potentially due to residual IFN-β and compensatory adaptive immunity.

## Teaser

Campbell et al. describe seven IRF7-deficient patients with severe respiratory viral infection. Combining genetic, immunological, and clinical investigation, they highlight the surprisingly narrow disease susceptibility of IRF7 deficiency and reveal potential compensatory immunological mechanisms, including IFN-β and adaptive immunity.

## Graphical Abstract

**Figure:**
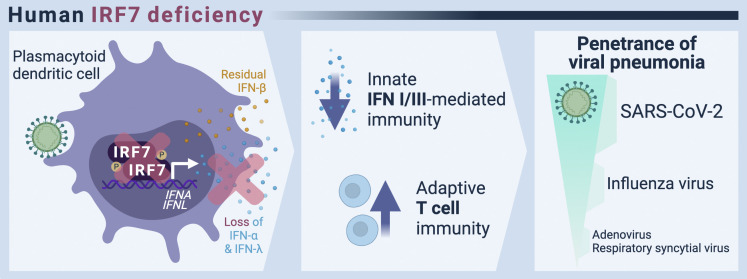

## Introduction

Human type I IFN–mediated immunity provides an intrinsic, innate first line of defense against invading viruses (Lazear et al., 2019; Schneider et al., 2014). The 17 type I IFN genes encode 13 forms of IFN-α, IFN-β, IFN-ε, IFN-κ, and IFN-ω. All nucleated cells can produce IFN-β upon sensing viral infection, and this contributes to the induction of other type I IFNs. During viral infections, plasmacytoid dendritic cells (pDCs) produce large quantities of IFN-α and -ω (Cella et al., 1999; Reizis, 2019; Siegal et al., 1999). IFN-κ and IFN-ε are preferentially expressed in the skin and reproductive tract, respectively, and are three orders of magnitude less potent than IFN-α2 (Fung et al., 2013; Harris et al., 2018; LaFleur et al., 2001). Nucleated cells express type I IFN receptors, whereupon stimulation induces the transcription of type I IFN genes and other IFN-stimulated genes (ISGs), most of which promote antiviral immunity (Schoggins, 2019). Notably, however, human pluripotent stem cells constitutively express ISGs and display attenuated induction of ISGs upon type I IFN stimulation (Hong and Carmichael, 2013; Wu et al., 2018).

*Ifnar1* or *Ifnar2* knockout mice that lack the type I IFN receptor are susceptible to many experimental infections, but an unexpected pattern is emerging in humans, with the corresponding deficits seeming to confer vulnerability to a narrower range of viruses (Duncan et al., 2021; Meyts and Casanova, 2021). Autosomal recessive (AR) IFNAR1 and IFNAR2 deficiencies have been reported in 16 and 9 patients, respectively (Abolhassani et al., 2022; Bastard et al., 2022; Bastard et al., 2021; Duncan et al., 2015; Duncan et al., 2022; Gothe et al., 2020; Hernandez et al., 2019; Zhang et al., 2020). Patients with inherited STAT2 or IRF9 deficiency lack ISG factor 3 (ISGF-3)—a transcription factor complex consisting of STAT1, STAT2, and IRF9 that normally induces ISG expression in response to type I and type III IFNs—but these patients have a similarly narrower range of viral susceptibility (Alosaimi et al., 2019; Hambleton et al., 2013; Hernandez et al., 2018; Moens et al., 2017).

Patients with an apparent complete absence of type I IFN immunity (IFNAR1, IFNAR2) or of ISGF-3 (STAT2, IRF9) are prone to adverse reactions to live attenuated viral vaccines, such as yellow fever virus 17D (YFV-17D) and the measles, mumps, and rubella virus (MMR) vaccine, and are also susceptible to HSV-1, encephalitis, and critical influenza or COVID-19 pneumonia (Abolhassani et al., 2022; Alosaimi et al., 2019; Bastard et al., 2021; Bastard et al., 2022; Duncan et al., 2015; Duncan et al., 2022; Hambleton et al., 2013; Hernandez et al., 2019; Hernandez et al., 2018; Moens et al., 2017; Zhang et al., 2020). The clinical penetrance of such infections in patients with type I IFN deficiencies remains unclear. These patients seem to be otherwise normally resistant to a number of common viruses. By contrast, AR complete and partial STAT1-deficient patients, with impairments of both ISGF-3 and γ-activated factor and, thus, unresponsive to type I, II, and III IFNs, are prone to various viral and intramacrophagic infections, resulting in early-onset disease following a devastating course (Boehmer et al., 2020; Burns et al., 2016; Chapgier et al., 2006; Dupuis et al., 2003; Le Voyer et al., 2021; Sakata et al., 2020; Vairo et al., 2011).

IRFs are a family of transcription factors initially identified on the basis of their ability to promote type I IFN production (Miyamoto et al., 1988). IRF3 and IRF7 have been implicated in the transcription of type I IFN genes downstream from viral sensors, whereas other members of the IRF family promote the transcription of a subset of ISGs (e.g., IRF9) or regulate leukocyte development and differentiation (e.g., IRF8). These IRFs typically bind regulatory DNA elements similar to those bound by ISGF-3 (IFN-stimulated response element boxes). Studies of knockout mice have revealed a key role for IRF7 in the production of type I and III IFNs (Honda et al., 2005). These studies also showed that pDCs are the most potent type I and III IFN-producing cells, because of their markedly high levels of constitutive IRF7 expression (Colonna et al., 2004; Honda and Taniguchi, 2006; Liu, 2001; Reizis, 2019). *Irf7*-knockout mice are much more susceptible to fatal HSV-1, encephalomyocarditis virus, and influenza A virus (IAV) infections than either WT or *Irf3*-knockout mice, indicating a requirement for IRF7 in immunity to both DNA and RNA viruses (Hatesuer et al., 2017; Honda et al., 2005).

In humans, a 2.5-yr-old child with life-threatening influenza virus pneumonia was found to be compound heterozygous for loss-of-function (LoF) *IRF7* variants (Ciancanelli et al., 2015). This case confirmed the requirement for IRF7 for the induction of all type I and III IFNs, with the exception of IFN-β. More recently, two unrelated and previously healthy adults presented with life-threatening COVID-19 pneumonia due to inherited IRF7 deficiency (Zhang et al., 2020). Surprisingly, neither of these patients presented any clinically severe viral infections until the ages of 49 and 50 yr. This raises important questions regarding the requirement for IRF7-dependent type I and III IFNs for human immunity to viruses. With brief descriptions of only three patients, the range of severe viral infections and the individual penetrance of each viral infection in patients with inherited IRF7 deficiency remained unclear. Here, we studied the genetic, immunological, and clinical features of an international cohort of seven patients from six kindreds and five ancestries with AR IRF7 deficiency, including the three previously described patients.

## Results and discussion

### Patients with biallelic rare *IRF7* variants

In 2015, we reported AR IRF7 deficiency in a single patient with life-threatening influenza pneumonia (P1, F410V;Q421X; Ciancanelli et al., 2015). In 2020, we reported another two patients with AR IRF7 deficiency (P2, P364AfsX38;P364AfsX38 and P3, D117N;M371V) and life-threatening COVID-19 pneumonia, providing proof-of-principle that critical influenza and COVID-19 pneumonia can be allelic (Casanova and Abel, 2021; Zhang et al., 2022; Zhang et al., 2020). We have now performed whole-exome sequencing (WES) or whole-genome sequencing on the COVID Human Genetic Effort cohort of 927 adult patients with critical COVID-19 pneumonia (Casanova et al., 2020). We have also analyzed a cohort of 107 patients with severe influenza pneumonia. We searched for patients with biallelic very rare variants of *IRF7* (minor allele frequency [MAF] <0.001).

**Figure 1.**
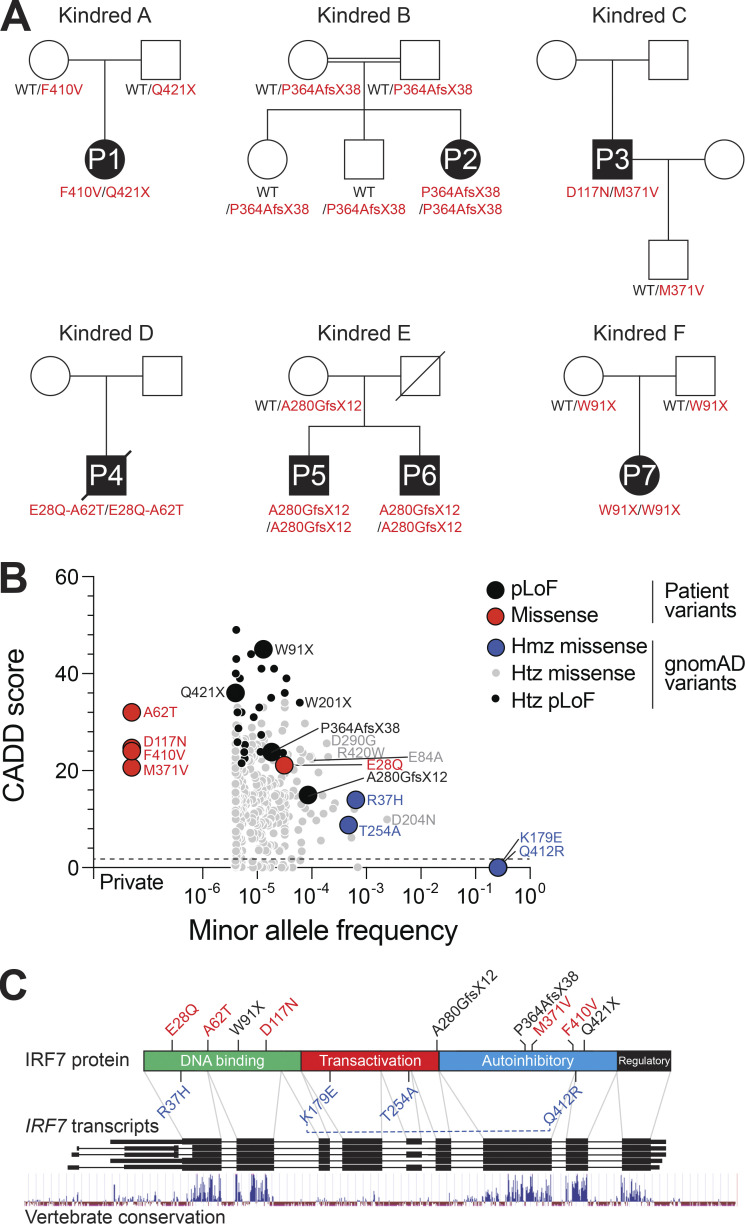
**Patients with biallelic *IRF7* variants. (A)** Pedigrees of the six kindreds containing seven patients with life-threatening viral infections (P1–P7) bearing rare biallelic *IRF7* variants. Solid black symbols indicate patients with critical viral infections. The *IRF7* genotype is indicated under each symbol. **(B)** The plot depicts the population frequency of *IRF7* missense and pLoF variants (gnomAD v2.1.1) against CADD score (v1.6, GRCh37). Symbols indicate a total of 463 variants, 4 identified exclusively in patients and 459 present in the gnomAD database. The patient-derived variants reported in this study are highlighted, with pLoF and missense variants colored black and red, respectively. The population-derived homozygous *IRF7* missense variants are highlighted in blue. **(C)** Schematic representation of IRF7. The lower part represents the genomic organization of the *IRF7* locus, with black rectangles indicating the exons of the gene according to different transcripts. Below, a track indicates vertebrate nucleotide conservation across the *IRF7* locus. The upper part shows the primary protein domain structure of IRF7. The N-terminal portion contains an α-helical DNA binding domain, followed by domains implicated in transactivation, autoinhibition, and regulation, as indicated. The positions of the patient-derived biallelic and population-derived homozygous *IRF7* variants are indicated. A blue dotted line indicates the linkage of the IRF7 K179E and Q412R variants. Hmz, homozygous; Htz, heterozygous.

In addition to the three previously reported patients with critical influenza or COVID-19 pneumonia, we identified one patient (P4) homozygous for combined *IRF7* (E28Q;A62T) variants and two patients (P5 and P6) homozygous for an *IRF7* A280GfsX12 variant presenting with critical and severe COVID-19, respectively. We also identified a patient (P7) homozygous for *IRF7* W91X in the cohort of patients with severe influenza. In total, we identified seven patients from six kindreds with life-threatening respiratory viral infections: five homozygotes and two compound heterozygotes. Cumulatively, we identified nine biallelic patient-associated *IRF7* variants (Fig. 1, A and B; and Table S1), five of which have previously been shown experimentally to be biochemically deleterious (Ciancanelli et al., 2015; Zhang et al., 2020). All of these *IRF7* variants had combined annotation-dependent depletion (CADD) scores—a measurement of the deleteriousness of the variant used to prioritize causal variants in genetic analyses (Kircher et al., 2014)—greater than 10 and above the mutation significance cutoff (Itan et al., 2016).

### Homozygous *IRF7* variants in public databases

We searched for nonsynonymous or splice site variants of *IRF7* present in the homozygous state in at least one individual reported in the public gnomAD database (v2.1.1, *IRF7* transcript ENST00000397574.2; Karczewski et al., 2020). Four missense variants were found: *IRF7* Q412R (global MAF = 0.26), K179E (MAF = 0.26), R37H (MAF = 0.0006), and T254A (MAF = 0.0005; Table S1). *IRF7* Q412R and K179E were in complete linkage disequilibrium (D′ and *r*^2^ = 1) in all 1,000 Genomes populations. We therefore considered them to form a single allele, representing a common *IRF7* (Q412R;K179E) variant. For the 13 biallelic *IRF7* variants, including nine identified in patients, with the other four identified in gnomAD, we found that only two of the common gnomAD variants did not have CADD scores above the mutation significance cutoff (Fig. 1 B and Table S1; Itan et al., 2016; Zhang et al., 2018). The 13 *IRF7* variants were located in different segments of the *IRF7* coding sequence. There were four nonsense variants predicted to be LoF (pLoF) and nine missense variants (Fig. 1 C). Previous functional evaluations have shown the *IRF7* R37H and T254A variants to be neutral, whereas the *IRF7* F410V, Q421X, D117N, M371V, and P364AfsX38 variants were found to be deleterious (Ciancanelli et al., 2015; Zhang et al., 2020). No functional evaluations have been performed for the other six variants.

### Population genetics of the *IRF7* gene

In addition to the four homozygous variants, there were 459 nonsynonymous coding and 13 splice donor or acceptor site variants of *IRF7* present in the heterozygous state in the gnomAD database (v2.1.1, *IRF7* transcript ENST00000397574.2). The nonsynonymous coding variants comprised 389 missense, 9 in-frame deletion or insertion, and 60 frameshift or stop-gain variants. The most common (MAF >0.01) missense variants were Q412R and K179E, and another 16 variants had a MAF >0.0001 (Table S1). Seven of these variants—IRF7 R37H, R131Q, G214E, G247R, T254A, S285T, and R423P—were found to be neutral in functional tests (Ciancanelli et al., 2015; Zhang et al., 2020). Four of the IRF7 variants that had not previously been tested—IRF7 E84A, D290G, and R420W—had CADD scores >20 (CADD v1.6-GRCh37; Table S1), suggesting a likelihood of these variants being damaging and warranting functional investigations. The stop-gain, splice donor, or acceptor variants were all very rare (MAF <0.0001). The *IRF7* A280GfsX12 variant, found in the homozygous state in two patients with critical and severe COVID-19 (P5 and P6, respectively), was the most frequent *IRF7* pLoF variant in gnomAD (MAF = 0.00009). It is located in coding exon 6 of the gene and had a surprisingly low CADD score (10.1). *IRF7* exons 3–6 encode a transactivation domain that has been poorly conserved during evolution, contrasting with the other exons encoding the DNA-binding, autoinhibitory, and regulatory domains of the protein (Fig. 1 C). Furthermore, exons 3, 5, 6, and 7 are symmetric, potentially facilitating the modular exclusion of exons 3–6 or of exon 6 alone, which would allow in-frame protein translation. The individuals heterozygous for the *IRF7* A280GfsX12 variant in gnomAD were predominantly of Swedish or Finnish ancestry (MAF 0.0007 and 0.0002, respectively). The high regional prevalence of this variant calls into question its putative deleteriousness and pathogenicity.

### Expression and function of *IRF7* variants in vitro

**Figure 2.**
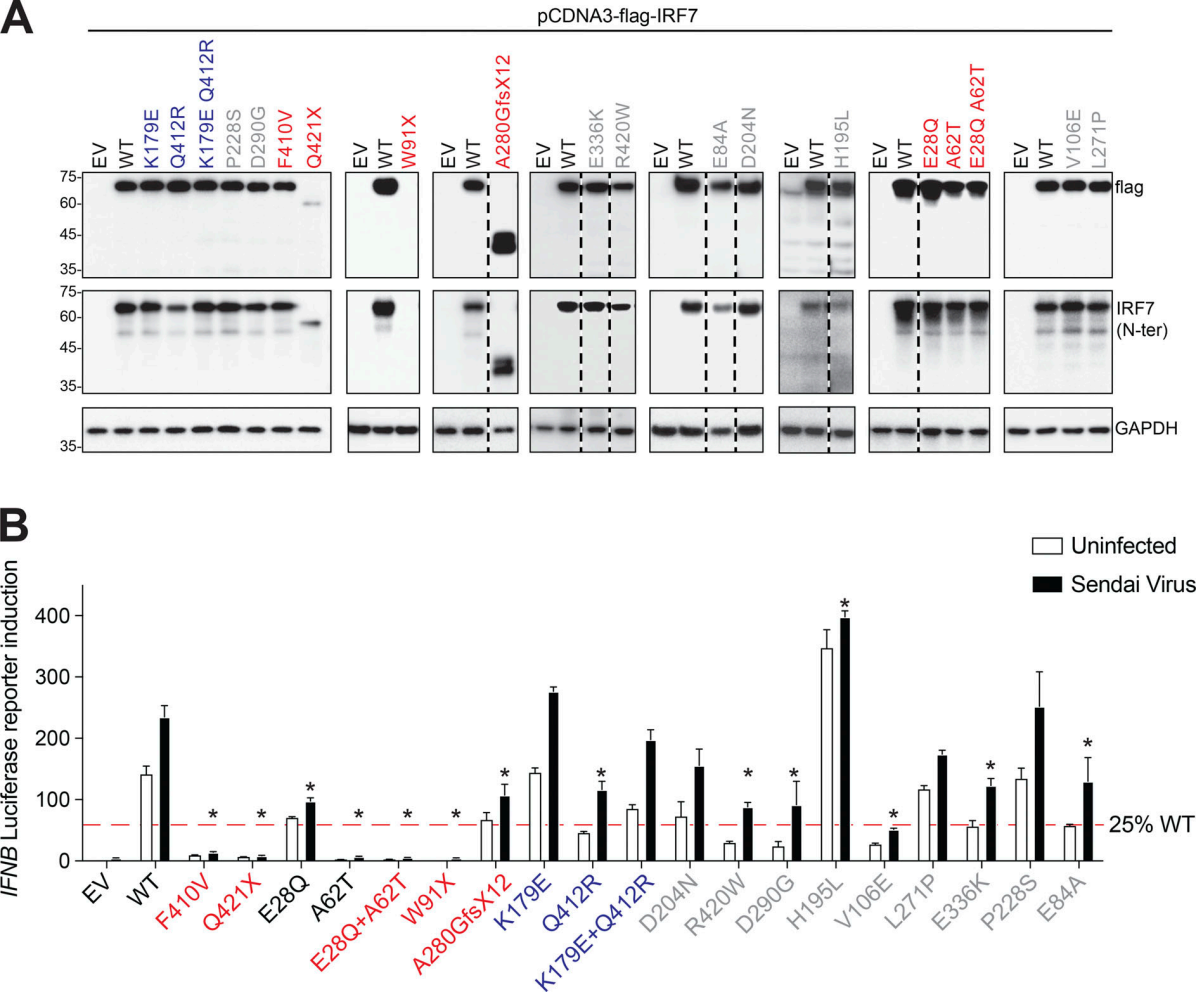
**Expression and activity of novel *IRF7* variants. (A)** HEK293T cells were transiently transfected with WT or mutant forms of *IRF7*. IRF7 levels were assessed by Western blotting with antibodies against the N-terminus of IRF7 or an N-terminal FLAG tag. GAPDH was used as a loading control. Representative immunoblots from at least three independent experiments are shown. EV, empty vector. **(B)** HEK293T cells were transiently transfected with WT or mutant forms of *IRF7*, together with an IFN-β luciferase reporter and a constitutively expressed reporter. Cells were either left untreated or infected with Sendai virus for 24 h before the assessment of normalized luciferase activity. The significance of differences between variants and the WT (mean ± SEM of *n* ≥ 3 independent experiments) was determined by two-way ANOVA (*, P < 0.05). Source data are available for this figure: SourceData F2.

We first assessed the expression and function of the six newly discovered patient-derived *IRF7* variants and the four missense *IRF7* variants identified in a homozygous state in gnomAD. By transfecting HEK293T cells with plasmids encoding the different *IRF7* variants, we showed that the missense variants, including the combined (E28Q;A62T) and (K179E;Q412R) variants, were expressed normally (Fig. 2 A). By comparison, the plasmids encoding A280GfsX12 and W91X yielded proteins of a lower molecular weight or resulted in a loss of expression, respectively. We assessed transcriptional activity by cotransfecting HEK293T cells with plasmids encoding the various *IRF7* variants and a luciferase reporter plasmid containing the regulatory sequence of *IFNB*, with or without Sendai virus infection, which induces type I IFN expression. The two previously reported *IRF7* LoF variants identified in P1, F410V and Q421X, served as controls. We found that IRF7 E28Q had lower levels of activity than the WT IRF7, whereas IRF7 A62T and the combined (E28Q;A62T) variants (as seen in P4) were completely defective (Fig. 2 B). IRF7 W91X (from P7) was LoF, consistent with the DNA-binding domain of IRF7 being located at the N-terminus of the protein (aa 1–150). By contrast, IRF7 A280GfsX12, from P5 and P6, was less active than IRF7 WT protein, but activity was not completely absent. Interestingly, the two common variants, K179E and Q412R, displayed higher and lower levels of activity, respectively, than WT IRF7, when tested separately (Fig. 2 B). As these two variants can be considered to constitute a single allele due to their strict linkage disequilibrium, we also tested their activity when combined into a single allele. Under these conditions, IRF7 (K179E;Q412R) had normal activity, similar to that of WT IRF7. Thus, the three newly described homozygous *IRF7* variants in our four patients with severe respiratory viral infections are deleterious, whereas variants found in the homozygous state in the general population are neutral. Finally, we tested all additional variants identified in the heterozygous state in the general population with a MAF >0.0001, some of which had relatively high CADD scores (Table S1). IRF7 D204N, P228S, and L271P were neutral, whereas E84A, V106E, D290G, E336K, and R420W had low levels of activity. IRF7 H195L had higher levels of activity than the WT IRF7. Collectively, these results are consistent with *IRF7* being under moderate negative selection, like other genes underlying AR inborn errors of immunity (pLI = 0; consensus negative selection [CoNeS] = 0.212; supervised CoNeS [SCoNeS] = 0.858), with a cumulative pLoF = 0.001 (Rapaport et al., 2021).

### IRF7 expression and function in patients’ cells

**Figure 3.**
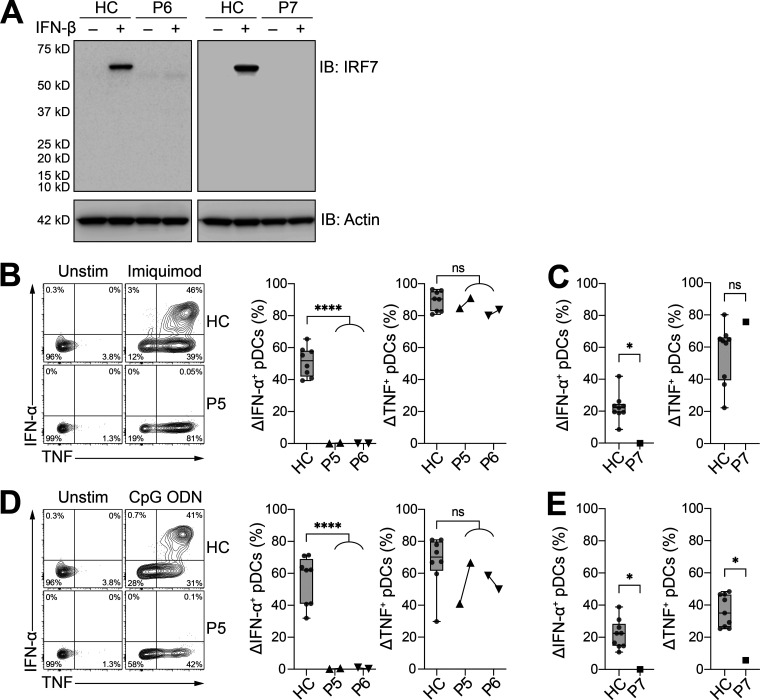
**Patients with the newly discovered *IRF7* variants do not produce IRF7 protein or IFN-α upon pDC stimulation. (A)** Protein levels for IRF7 in PBMCs with and without IFN-β stimulation for 24 h; comparison of patients and healthy controls (HC). Actin staining was used as a loading control. Representative immunoblots (IB) from single independent experiments per patient are shown. **(B and C)** Frequency of IFN-α– and TNF-producing pDCs (live Lin^–^CD11c^–^HLA-DR^+^CD303^+^CD123^+^) after 6 h of stimulation with imiquimod for fresh (B) and thawed (C) PBMCs. **(D and E)** Frequency of IFN-α– and TNF-producing pDCs (live Lin^–^CD11c^–^HLA-DR^+^CD303^+^CD123^+^) after 6 h of stimulation with CpG ODN for fresh (D) and thawed (E) PBMCs. For P5 and P6, cells were assessed at two independent time points 6 mo apart. **(A–E)** All data are presented as the frequency of responding cells minus the frequency of the corresponding unstimulated controls. Box plots are bound by the 25th and 75th percentiles. The median is marked, and the whiskers indicate the minimum and maximum. Individual values are plotted. Unpaired *t* tests were performed to compare healthy controls (HC; *n* = 8–9) with patients. *, P < 0.05; ****, P < 0.0001. Source data are available for this figure: SourceData F3.

We investigated the effect of the newly described *IRF7* variants in patients, by first assessing the induction of IRF7 expression in peripheral blood mononuclear cells (PBMCs) stimulated with IFN-β. Samples were available from kindreds E (P6) and F (P7). Despite the production of IRF7 fragments of lower molecular weight in ectopic expression systems of *IRF7* A280GfsX12 (Fig. 2 A), we detected no IRF7 in the cells of patients P6 or P7 after stimulation with IFN-β (Fig. 3 A). We previously tested pDCs from two other IRF7-deficient patients and showed that IRF7-deficient pDCs could produce some IFN-β upon viral or TLR stimulation, but none of the other type I or III IFNs (Ciancanelli et al., 2015; Zhang et al., 2020). We evaluated IRF7 activity in patients P5, P6, and P7 by stimulating PBMCs with TLR7 (imiquimod) or TLR9 (CpG oligodeoxynucleotide [ODN]) agonists, which are known to induce strong IFN-α and TNF production by pDCs (Cella et al., 1999; Reizis, 2019; Siegal et al., 1999). In patients P5 and P6, pDCs did not produce IFN-α in response to TLR7 or TLR9 agonists (Fig. 3, B and D). By contrast, the TLR agonists induced strong TNF production by pDCs, demonstrating a specific requirement of IRF7 for the production of IFN-α, but not other proinflammatory cytokines, such as TNF. Both P5 and P6 had normal numbers of pDCs in peripheral blood, with 36 and 73 cells/μl of whole blood, respectively (healthy reference range: 12–178 cells/μl, median 64 cells/μl, *n* = 38). Similarly, pDCs from P7 did not produce IFN-α in response to TLR7 or TLR9 agonists (Fig. 3, C and E).

**Figure S1.**
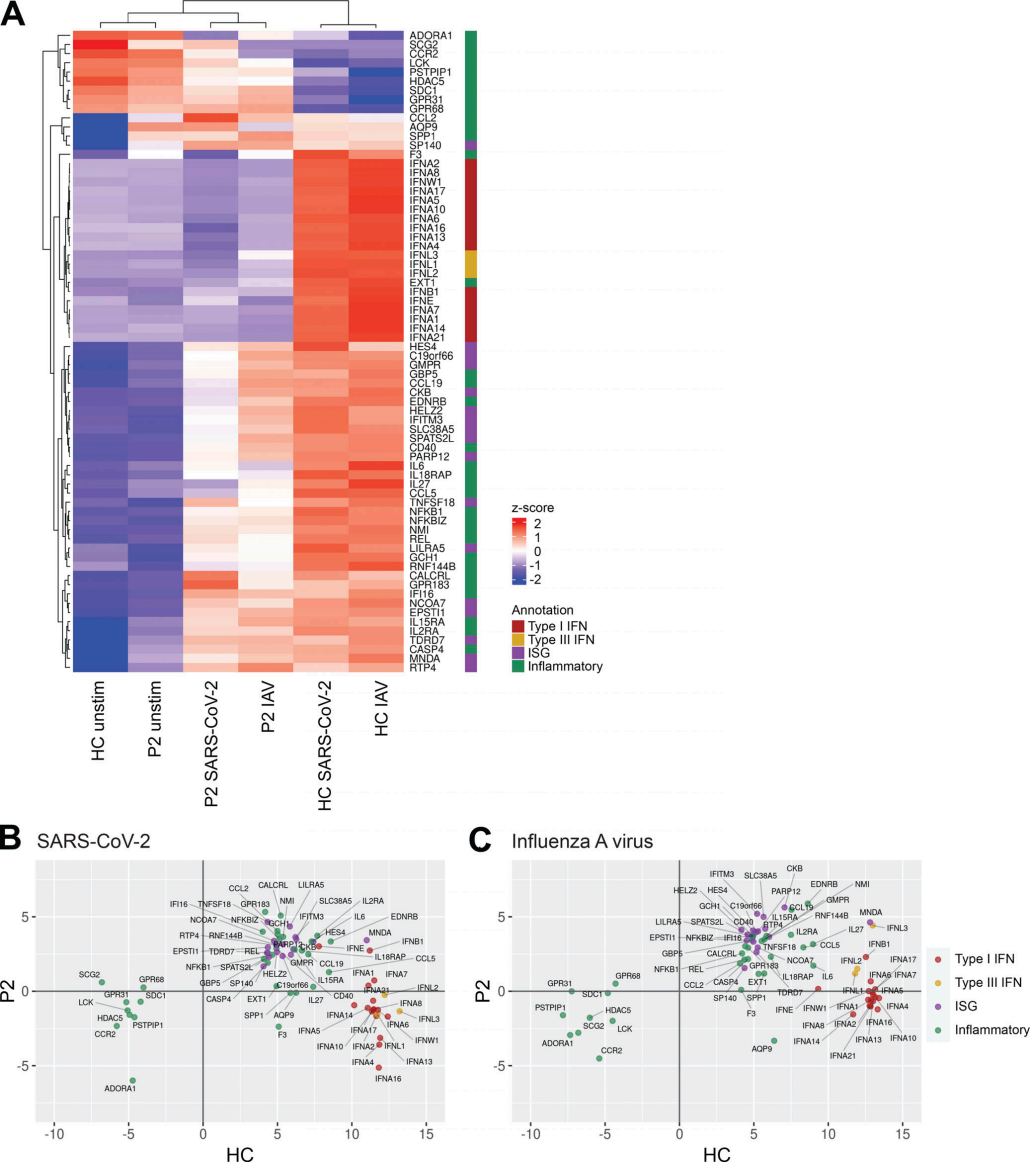
**IFN and inflammatory responses in IRF7-deficient pDCs challenged with SARS-CoV-2 and IAV.** RNA-seq analysis of isolated pDCs from P2 and a healthy control (HC) either unstimulated or cultured with SARS-CoV-2 or IAV. **(A–C)** Genes in the annotated interest groups with expression >2.5-fold higher or lower in P2 vs. HC after viral culture were plotted as a heatmap of expression z-score (A) and expression fold-change in P2 vs. HC (B and C). Data are representative of a single experiment and reanalyzed from Zhang et al. (2020).

**Figure S2.**
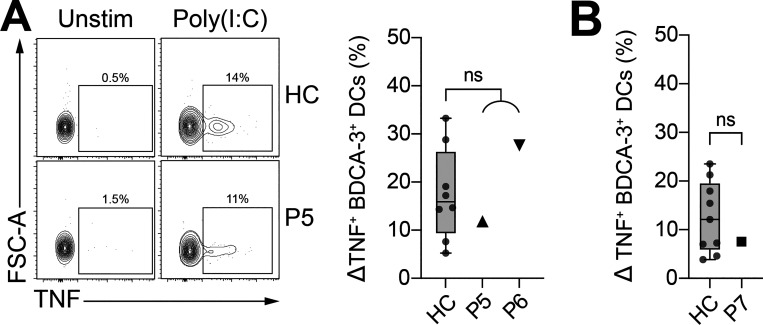
**Patients with newly discovered deleterious *IRF7* variants produce normal levels of TNF after the TLR3-mediated stimulation of BDCA3**^**+**^ **DCs. (A and B)** Frequency of TNF-producing BDCA3^+^ DCs (live Lin^–^CD123^–^HLA-DR^+^CD141^+^) after 6 h of stimulation with poly(I:C), for fresh (A) and thawed (B) PBMCs. All data are presented as the frequency of responding cells minus that of the respective unstimulated controls. Box plots are bound by the 25th and 75th percentiles. The median is marked, and the whiskers indicate the maximum and minimum. Individual values are plotted. Unpaired *t* tests were performed to compare healthy controls (HC; *n* = 8–9) and patients. FSC, forward scatter.

Of note, the transcriptional responses of P2 pDCs to SARS-CoV-2 and IAV infection were previously reported (Zhang et al., 2020). Further analyses of these data reveal that pDCs from P2 failed to respond to viral stimulation, in terms of the induction of type I and III IFNs transcripts, with the exception of *IFNB1* mRNA that was weakly induced (Fig. S1). Patient PBMCs were also treated with the TLR3 agonist poly(I:C), which stimulates TNF expression by BDCA-3^+^ DCs (Jongbloed et al., 2010). No significant difference in TNF production following TLR3 stimulation was observed between the patients and healthy controls, as expected (Fig. S2). Overall, PBMCs from patients with homozygous *IRF7*-truncating W91X and A280GfsX12 variants had no IRF7 protein and displayed defective IFN-α production by pDCs. The results of these physiological cellular assays thus indicate that the *IRF7* A280GfsX12 variant, which was hypomorphic when tested by overexpression in HEK-293T cells, is actually an LoF variant when constitutively expressed in pDCs, consistent with critical or severe COVID-19 pneumonia in both patients from kindred E (P5 and P6).

### Clinical characteristics of IRF7-deficient patients

All patients with biallelic IRF7 LoF variants presented clinically with severe viral respiratory infections (Table 1). The patients originated from five different countries: France (P1), Italy/Belgium (P2), Turkey (P3), Iran (P4), Sweden (P5 and P6), and Belgium (P7). Detailed descriptions of the clinical presentations of P4–P7 are provided in the supplementary material, with an update of the clinical presentations of the previously reported P1–P3. Age at clinical presentation ranged from 6 mo to 50 yr. Patients were healthy before presenting with severe infections with respiratory viruses: IAV (P1 and P6), SARS-CoV-2 (P2–P5), and respiratory syncytial virus (RSV; P7), all of which are single-stranded RNA viruses. Five patients were hospitalized for a single severe viral infection, whereas two suffered multiple severe viral infections. Patient P6 was initially hospitalized with IAV infection in adolescence before requiring oxygen treatment for severe COVID-19 in 2020, and patient P7 first presented with RSV at 6 mo of age and has since been treated for severe, consecutive IAV and adenovirus infections. Prophylactic measures, such as annual influenza vaccination (P1 since 2014; P5, P6, and P7 since 2021) and COVID-19 vaccination (P1, P2, P3, P5, P6, and P7 in 2021), were implemented after molecular diagnosis.

**Table 1.** Genetic, demographic, and clinical features of IRF7-deficient patients

Feature	P1	P2	P3	P4	P5	P6	P7
***IRF7* variants (NM_001572)**	p.(Phe410Val); p.(Gln421Ter)	p.(Pro364AlafsX38); p.(Pro364AlafsX38)	p.(Asp117Asn); p.(Met371Val)	p.(Glu28Gln;Ala62Thr); p.(Glu28Gln;Ala62Thr)	p.(Ala280GlyfsX12); p.(Ala280GlyfsX12)	p.(Ala280GlyfsX12); p.(Ala280GlyfsX12)	p.(Trp91Ter); p.(Trp91Ter)
**Age at onset**	2.5 yr	49 yr	50 yr	29 yr	38 yr	31 yr	6 mo
**Ancestry (Residence)**	France	Italy (Belgium)	Turkey	Iran	Sweden/Finland (Sweden)	Sweden/Finland (Sweden)	Belgium
**Viral susceptibility**	Influenza	SARS-CoV-2	SARS-CoV-2	SARS-CoV-2	SARS-CoV-2	Influenza; SARS-CoV-2; TBE virus	RSV; influenza; adenovirus
**BMI (kg/m** ^**2**^ **)**		30.0	27.3	29.4	23.4	22.0	
**Risk factors**		Obesity	None		None	None	
**Outcome**	Alive and well at age 14 yr	Alive and well at age 50 yr	Alive and well at age 51 yr	Died from COVID-19 at age 29 yr	Alive and well at age 39 yr	Alive and well at age 32 yr	Alive and well at age 5 yr
**Vaccinations**	Influenza since 2014; COVID-19 since 2021			Not applicable	Influenza since 2021; COVID-19 since 2021	Influenza since 2021; COVID-19 since 2021	Influenza since 2021; COVID-19 since 2021
**Reference**	Ciancanelli et al., 2016	Zhang et al., 2020	Zhang et al., 2020				

### Adaptive antiviral T cell immunity in IRF7-deficient patients

**Figure 4.**
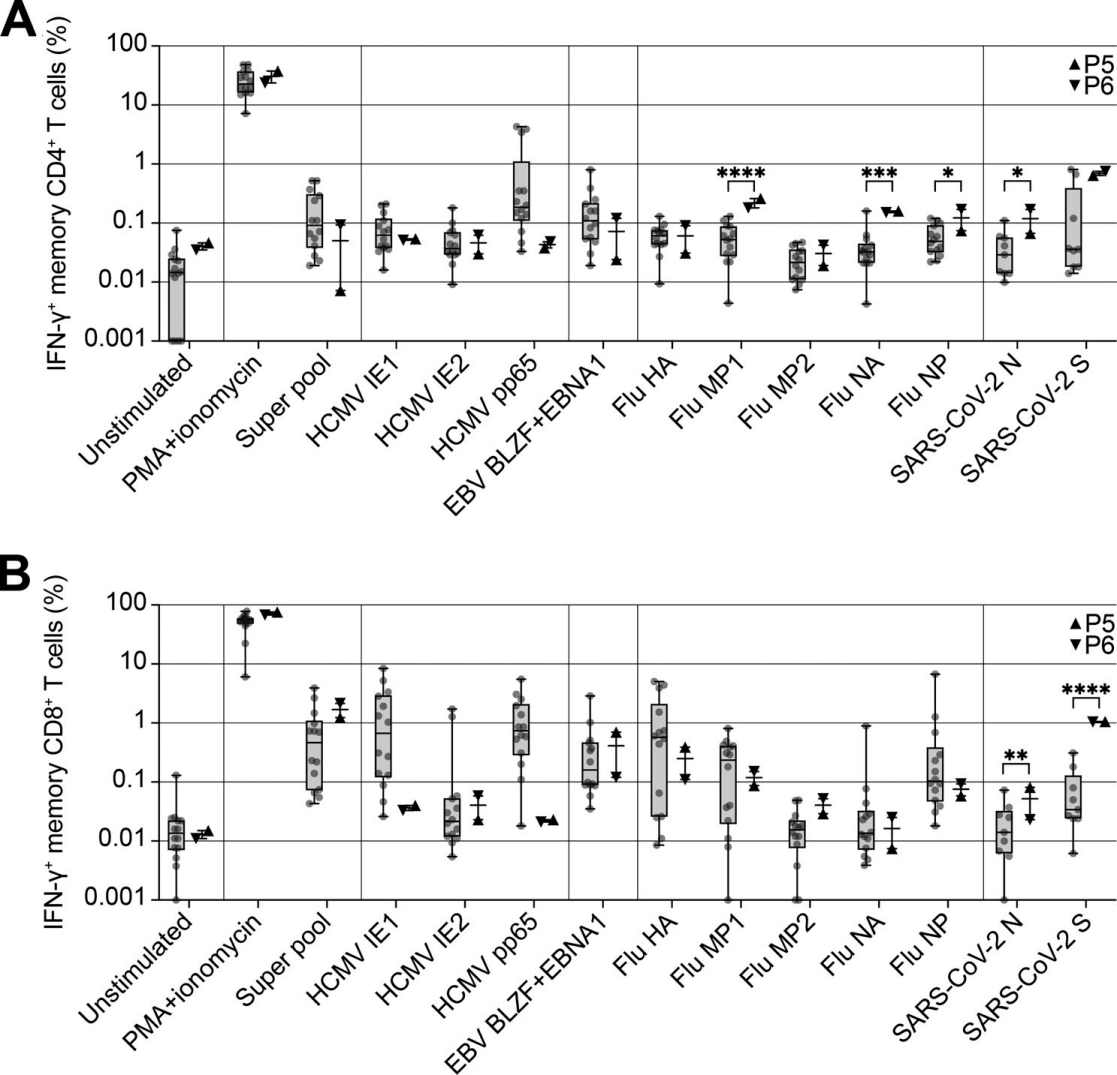
**IRF7-deficient patients have enhanced CD4**^**+**^ **T cell responses to influenza and coronaviruses. (A and B)** Frequency of IFN-γ–producing memory CD4^+^ (live Lin^–^CD3^+^CD4^+CD8–^CCR7^–^CD95^+^; A) or CD8^+^ (live Lin^–^CD3^+CD8+^CD4^–^CCR7^–^CD95^+^; B) T cells after stimulation with peptides or PMA + ionomycin for 6 h. Unstimulated controls are shown on the left of the graph. Box plots are bound by the 25th and 75th percentiles. The median is marked, and the whiskers indicate the maximum and minimum. Individual values are plotted. Unpaired *t* tests were performed to compare healthy controls (HC; *n* = 9–14) and patients (P5 and P6). *, P < 0.05; **, P < 0.01; ***, P < 0.001; ****, P < 0.0001.

**Figure S3.**
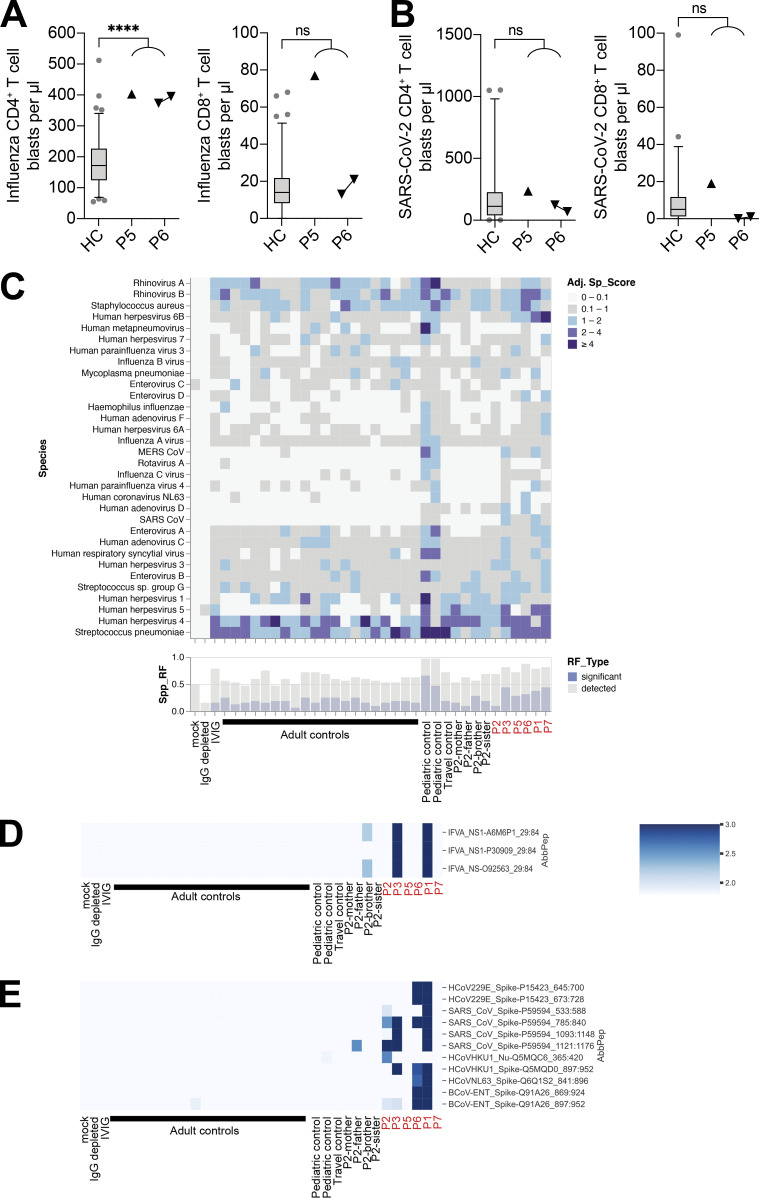
**Large numbers of influenza-specific CD4**^**+**^ **T cell blasts in IRF7-deficient patients. (A and B)** Whole-blood CD4^+^ (CD3^+^CD4^+^) and CD8^+^ (CD3^+^CD4^–^) T cell blast responses to influenza split-virus vaccine (A) or SARS-CoV-2–inactivated virus (B) after 6 d of stimulation; comparison of healthy controls (HC, *n* = 91 [A] or *n* = 46 [B]) with patients. For P6, cells were assessed at two independent time points 1 yr apart. Box plots are bound by the 25th and 75th percentiles with the median marked. The whiskers indicate the 5th and 95th percentiles, and outlier values are plotted. Unpaired *t* tests with Welch’s correction were performed to compare the HC and patient groups. ****, P < 0.0001. **(C–E)** VirScan assay showing the presence of antibodies against viruses in the serum samples from patients and controls, with the analysis focusing on IAV (D) and coronaviruses (E). Data are representative of a single experiment. Adj Sp_score, adjusted species score; spp_RF, significant species response frequency; AbbPep, abbreviation of peptide (SpeciesName_ProteinName_UniprotID_start_end).

T cell responses are a hallmark of adaptive immunity to viral infections (Bradley and Thomas, 2019). We therefore quantified memory T cell responses in patients P5 and P6 in response to stimulation with peptides from a range of common viruses. The IFN-γ responses of the patients’ cells were similar to those of healthy controls following stimulation with peptides from human herpesviruses, human cytomegalovirus (HCMV) and EBV (Fig. 4, A and B). Interestingly, both patients had significantly higher frequencies of memory CD4^+^ T cells responding to three of the five IAV peptides tested (MP1, NA, and NP) than the controls (Fig. 4 A). Furthermore, the patients had significantly higher frequencies of SARS-CoV-2–responding CD4^+^ and CD8^+^ memory T cells than healthy donors, including individuals with a history of mild COVID-19. Both patients were hospitalized for COVID-19, but only P6 had previously suffered from severe influenza, despite both P5 and P6 having high frequencies of IAV-specific CD4^+^ memory T cells. Notably, the analyzed samples were collected from the patients before vaccination against influenza and COVID-19. We also analyzed T cell responses in patients P5 and P6 after the stimulation of whole blood with influenza inactivated split-virus vaccine or whole inactivated SARS-CoV-2 virus, quantifying T cell blasts after 6 d of stimulation. Consistent with the frequency of CD4^+^ memory T cells responding to IAV peptides (Fig. 4 A), P5 and P6 had significantly stronger CD4^+^ T cell blast responses to influenza virus vaccine stimulation than healthy controls (Fig. S3 A). In response to SARS-CoV-2, both P5 and P6 had CD4^+^ and CD8^+^ T cell responses within the range observed for healthy controls convalescing from COVID-19 (Fig. S3 B).

### Adaptive antiviral B cell immunity in IRF7-deficient patients

We assessed the humoral responses to viruses by investigating the exposure of the patients to viruses with VirScan (Xu et al., 2015). Samples were collected from the patients before vaccination against influenza and COVID-19. We found that the patients were seropositive for pathogens commonly found in age-matched controls, showing that they were able to control most common pathogens, including rhinoviruses, herpesviruses, enteroviruses, *Streptococcus pneumoniae*, and *Mycoplasma pneumoniae* (Fig. S3 C). In analyses focusing on antigens specific to IAV, P1 and P3 were seropositive for the IAV nonstructural 1 (NS1) protein (Fig. S3 D). In analyses focusing on antigens specific to coronaviruses, P1, P2, P3, and P6 were seropositive for SARS-CoV-2, reflecting their known history of COVID-19 (P2, P3, and P6) and vaccination (P1). Surprisingly, P5 was seronegative for all four SARS-CoV-2 spike protein–specific antigens. P1 and P6 were also seropositive for antigens specific to common coronaviruses, such as 229E and NL63, suggesting that they were able to control less virulent coronaviruses (Fig. S3 E). In patients P5 and P6, we confirmed by classic serology that both patients had mounted antibody responses to EBV, HSV, measles virus, mumps virus, and rubella virus. Patients P1 and P7, who did not present with clinical COVID-19, have been vaccinated with three and two doses, respectively, of the BNT162b2 mRNA vaccine. P1 responded by producing antibodies against the SARS-CoV-2 spike protein, as demonstrated by the VirScan analysis (Fig. S3 E). P7 also developed antibodies against the spike protein after vaccination (anti-S IgG level 1 mo after the second dose: 20,195 AU/ml [cutoff > 5 AU/ml]). Thus, IRF7-deficient patients can mount robust adaptive responses to viruses and to mRNA vaccines, providing protection against severe infections. Moreover, these patients were found to have been infected with a wide range of common viruses, which they controlled normally.

### Impaired type I and III IFN expression in IRF7 deficiency predisposes to severe respiratory viral infections

Altogether, we report inherited IRF7 deficiency in seven patients from six kindreds in five countries. All nine alleles were hypomorphic when tested by overexpression in HEK293T cells (Fig. 2 B; Ciancanelli et al., 2015; Zhang et al., 2020); however, in primary cells from siblings P5 and P6, as well as P7, homozygous expression of two of the alleles conferred a complete functional defect. The patients from all six families thus suffer from a complete form of AR deficiency. Inherited IRF7 deficiency underlies at least four unusually severe respiratory viral infections, with influenza and COVID-19 being the most common and severe (Ciancanelli et al., 2016; Zhang et al., 2022). The mechanism of viral pneumonia in patients with IRF7 deficiency probably involves the disruption of type I and III IFNs in both pDCs and respiratory epithelial cells (Ciancanelli et al., 2015; Zhang et al., 2020), illustrated by undetectable blood IFN-α levels during COVID-19 infection (Zhang et al., 2020). Patients with TLR3 deficiency are prone to both influenza and COVID-19 pneumonia, perhaps because TLR3 regulates the basal type I IFN levels in respiratory epithelial cells, as it does in fibroblasts and cortical neurons (Gao et al., 2021; Lim et al., 2019; Zhang et al., 2020; Zhang et al., 2007). By contrast, patients with TLR7 deficiency have defective pDCs and seem to be normally resistant to influenza but highly vulnerable to COVID-19 (Asano et al., 2021). IRF7 deficiency affects both pDCs and epithelial cells and both type I and III IFNs. The intact type III IFN activity in IFNAR1-deficient patients with critical COVID-19 pneumonia suggests that the poor induction of type I IFN impairs host defense against SARS-CoV-2 in the respiratory tract of IRF7-deficient patients (Zhang et al., 2020). By contrast, critical influenza has been reported in only two of the 25 patients with IFNAR1 or IFNAR2 deficiency, suggesting that type III IFN may help to protect against influenza in most cases, accounting for the occurrence of critical influenza in IRF7-deficient patients, who have impaired production of both type I and III IFNs (Abolhassani et al., 2022; Bastard et al., 2022; Bastard et al., 2021; Duncan et al., 2015; Duncan et al., 2022; Gothe et al., 2020; Hernandez et al., 2019; Zhang et al., 2020).

### Variable disease penetrance in IRF7 deficiency

Penetrance is incomplete in IRF7-deficient patients, at least for RSV, adenovirus, and influenza, although we cannot be sure that the same serotypes infected other IRF7-deficient individuals. There is probably higher penetrance for COVID-19, as indicated by the two brothers (P5 and P6) manifesting life-threatening SARS-CoV-2 infection. Only one of the brothers presented clinically with influenza, but both had high influenza antibody titers and T cell responses indicative of prior infection. Most SARS-CoV-2 strains are more virulent and induce production of smaller amounts of type I IFN than seasonal influenza viruses (Blanco-Melo et al., 2020; Chu et al., 2020). The follow-up of existing IRF7-deficient patients and the molecular diagnosis of additional patients with this deficiency should shed further light on the clinical infectious disease spectrum and penetrance of IRF7 deficiency and the role of IRF7 in other noninfectious diseases (Bidwell et al., 2012; Zhao et al., 2017). *Irf7* knockout mice could be studied to provide more insight into the molecular and cellular basis of disease in IRF7-deficient patients, particular as such mice have a susceptibility to viral infections somewhat comparable to that of IRF7-deficient patients (Ciancanelli et al., 2016; Hatesuer et al., 2017). However, it is difficult to model natural human infections in inbred mice, because of the experimental nature of the infections in mice (Casanova and Abel, 2021). The combination of such studies with the use of induced pluripotent stem cell–derived pDCs and respiratory epithelial cells to model these infections in human cells could potentially elucidate some of the compensatory pathways reducing infectious disease penetrance (Sontag et al., 2017).

### Narrow spectrum of viral susceptibility in IRF7 deficiency compared with other type I IFN deficiencies

It is also surprising that IRF7-deficient patients are not prone to other viral infections repeatedly seen in many of the 25 patients with IFNAR1 or IFNAR2 deficiency reported to date, such as disseminated disease following MMR or YFV-17D vaccination and HSV-1 encephalitis (Abolhassani et al., 2022; Bastard et al., 2022; Bastard et al., 2021; Duncan et al., 2015; Duncan et al., 2022; Gothe et al., 2020; Hernandez et al., 2019). These viral susceptibilities, and predisposition to severe disease from additional human herpesviruses, are also common to patients with other core deficiencies of the type I IFN response, such as STAT1, STAT2, and IRF9 deficiencies (Duncan et al., 2021; Meyts and Casanova, 2021). These patients also have viral susceptibility profiles similar in some respects to those of IRF7-deficient patients, with select cases of severe disease caused by influenza viruses, RSV, and adenoviruses (Alosaimi et al., 2019; Hernandez et al., 2018; Le Voyer et al., 2021). However, it is striking that the IRF7-deficient patients have tolerated live-attenuated vaccines and so many common viruses, particularly those infecting tissues outside the respiratory tract. This finding also contrasts with those for *Irf7* knockout mice and in vitro studies with human IRF7-deficient cells, which have reported susceptibility to a broader range of viral infections (Hatesuer et al., 2017; Honda et al., 2005). Furthermore, pDCs from IRF7-deficient patients lack induction of type I and III IFNs, with the exception of IFN-β, whose induction was impaired but not abolished (Fig. S1; Ciancanelli et al., 2015; Zhang et al., 2020).

So, how can we account for the relatively narrow and pulmonary clinical phenotype of IRF7 deficiency relative to other type I IFN deficiencies (e.g., IFNAR1 deficiency)? A first hypothesis is that IFN-β may be involved, providing sufficient immunity to viruses in various tissues and organs. This would imply that IFN-β is insufficient to handle some respiratory viruses, at least in certain settings (a particularly virulent influenza strain, SARS-CoV-2, or a large inoculum of RSV or adenovirus). The lack of severe infections in IRF7-deficient patients after vaccination with live attenuated vaccines, especially MMR, which can cause severe infections in patients with deficiencies of STAT1, STAT2, IFNAR1, and IFNAR2, suggests that the amplification of type I IFN signaling mediated by IRF7 may be dispensable if the attenuated strains trigger stronger type I IFN responses than the WT virus (Poyhonen et al., 2019; Shingai et al., 2007). Furthermore, the patients’ narrow viral susceptibility may reflect the variable ways different viruses interact with human cells. Herpesviruses, for example—which were controlled normally by all IRF7-deficient patients—employ multiple immune evasion strategies to reduce type I IFN responses and establish latency (Garcia-Sastre, 2017; Hoffmann et al., 2015; Weekes et al., 2014). Conceivably, this process might limit the requirement for type I IFNs in the control of chronic herpesvirus infections.

Alternatively, or additionally, the patients’ myeloid and lymphoid cells may ensure more robust innate immunity than in patients with type I IFN receptor and signaling deficiencies. Our data suggest that IRF7-deficient patients can have particularly strong adaptive immunity to viruses. We show here that they have a robust CD4^+^ T cell response to IAV and SARS-CoV-2 (Fig. 4). Type I IFN has been implicated in both the promotion and limitation of T cell responses, the precise balance being determined by the timing of type I IFN exposure relative to T cell activation (Crouse et al., 2015). Low type I IFN levels, particularly in the bloodstream, may limit the natural killer cell–mediated killing of virus-specific T cells, thereby facilitating the establishment of larger memory T cell pools (Crouse et al., 2014). Established T cell responses may at least partly account for the absence of viral disease recurrence in these patients, at least at the time of reporting, even for respiratory tract infections, in particular SARS-CoV-2, which is likely the most virulent virus to have infected these patients.

In conclusion, characterization of the IRF7-deficient individuals identified to date suggests a relatively narrow susceptibility to critical infections with respiratory viruses, with some individuals displaying strong adaptive responses that may protect them against severe viral disease for decades. Residual IFN-β immunity might also contribute to host defense against viruses. Vaccination against common viral pathogens, including SARS-CoV-2, appears to be an effective intervention that can protect these patients from infectious disease.

## Materials and methods

### Patients

Samples were obtained from the probands, parents, and relatives, with written informed consent. The study was approved by the Swedish Ethical Review Authority (CovPID: 2020-01911), the Ethics Committee UZ Leuven (BIOKID: S63807), the Ethics Committee of Hôpital Erasme (P2020-203), the French Ethics Committee “Comité de Protection des Personnes,” the French National Agency for Medicine and Health Product Safety, the “Institut National de la Santé et de la Recherche Médicale,” in Paris, France (protocol no. C10-13); and the Rockefeller University Institutional Review Board in New York, NY (protocol no. JCA-0700).

### Case reports

#### Kindred A

The clinical presentation and progression of patient P1 have been described in detail elsewhere (Ciancanelli et al., 2015). Patient P1 has been vaccinated against influenza annually since 2014, with one or two doses, depending on the results of postvaccination serologic tests. She was also vaccinated with three doses of the COVID-19 BNT162b2 mRNA vaccine (Pfizer) in 2021. She has suffered no severe infections since 2011 and remains EBV seronegative.

#### Kindred B

Patient P2 is a Belgian woman born in 1971 to first-cousin parents of Italian origin. In March 2020, she presented with a flu syndrome, including fatigue, fever (39°C), nausea and vomiting, myalgia, and headache. She had no history of contact with a confirmed COVID-19 case, but her brother had developed flu-like symptoms lasting a few days in the previous week. Upon hospital admission 1 wk after onset, she was diagnosed with COVID-19 based on clinical presentation: fever, crackles on auscultation, dyspnea and hypoxemia (arterial oxygen saturation of 93% despite 6 liter/min oxygen support), a typical computerized tomography (CT) scan with bilateral basal condensation and widespread ground-glass opacities, and blood test results suggestive of an inflammatory syndrome (white blood cells: 13,680/mm^3^ with 84.5% polynuclear neutrophils, 11.3% lymphocytes, and 4.1% monocytes; C-reactive peptide [CRP]: 450 ng/liter). She also had high transaminase (aspartate aminotransferase: 53 IU/liter, alanine aminotransferase: 51 IU/liter) and γ-glutamyl transferase (157 IU/liter) levels. COVID-19 was confirmed by a positive SARS-CoV-2 PCR test on a nasopharyngeal swab, with negative results for IAV and IBV antigens.

The patient rapidly developed acute respiratory distress syndrome. She was admitted to the intensive care unit (ICU), intubated, placed on assisted ventilation, and treated with hydroxychloroquine and amoxicillin/clavulanic acid for 4 d. She was then shifted onto piperacillin/tazobactam and vancomycin. 1 wk after admission, her condition worsened owing to asthma exacerbation, and she suffered from hypoxemia and hypercapnia despite assisted ventilation. She was transferred to Hôpital Erasme for extracorporeal membrane oxygenation, which was maintained for 4 d. She was treated with hydroxychloroquine, lopinavir/ritonavir, piperacillin/tazobactam, corticosteroids, and bronchodilators. She developed subglottic edema, necessitating tracheostomy. Bacteriological analysis of bronchoalveolar lavage revealed superinfection with *Escherichia coli* and *Staphylococcus aureus* (cytology: leukocytes, alveolar macrophages, bronchial cells). The patient also developed a urinary tract infection with *E. coli*, for which she was treated with oxacillin for 10 d. She also carried multidrug-resistant *Enterococcus faecium*.

Progressive weaning from ventilation was initiated after 10 d on extracorporeal membrane oxygenation, and the tracheostomy was removed 10 d later. The patient was transferred to the pulmonology ward and discharged 6 wk after admission. She was still extremely malnourished and debilitated, requiring daily physiotherapy.

Relevant blood test results are as follows: blood type O+, maximum concentrations of 47 pg/ml for IL-6, 14,808 ng/ml for D-dimers, and 160 ng/liter for CRP. Persistent hepatic cytolysis and cholestasis were observed during the patient’s ICU stay. SARS-CoV-2 PCR remained positive until 1 wk before discharge. White blood cell count in remission: 8,700/mm^3^, with 44.5% neutrophils and 46.4% lymphocytes.

The patient had had asthma since the age of 30 yr, treated with inhaled ipratropium bromide and beclomethasone. She was overweight (body mass index [BMI] 30 kg/m^2^), with type 2 diabetes treated with metformin and tachycardia episodes treated with bisoprolol. Previous medical history included surgery for carpal tunnel syndrome and traumatic brain injury (with the fortuitous discovery of benign brain lesions).

The patient’s previous infectious history is as follows: The patient described recurrent infections since childhood. She underwent tonsillectomy at ∼9 yr of age and subsequently suffered four to five episodes of sinusitis and/or otitis per year until adulthood. These infections were considered viral, and therefore she rarely received antibiotics. She had four flu episodes, twice as a teenager and twice as an adult, with 7–10 d of high fever, fatigue, nausea and vomiting, myalgia, and dizziness. She had never been vaccinated against influenza. She had bronchitis with asthma exacerbation every year until a few years ago but had never had to be hospitalized. She developed hepatitis (digestive symptoms and icterus) in childhood, together with her siblings. She recalls suffering from mumps, varicella, and measles as a child, as well as numerous other infectious episodes.

She had not been vaccinated with Bacillus Calmette–Guérin. She received a preconception recall rubella vaccine and had anti-rubella IgG. Blood tests also revealed CMV exposure, with a normal IgG response.

The patient’s father is diabetic and has a heart condition. She has a sister living with the parents who has suffered from depression and underwent tonsillectomy. Her brother also underwent tonsillectomy and is in good health. The patient has three healthy daughters, aged 18, 22, and 26 yr.

#### Kindred C

Patient P3 is a Turkish man born in 1960 who contracted COVID-19 in April 2020. He was hospitalized and admitted to the ICU. He presented with flu-like symptoms, including cough, dyspnea, fever, headache, and myalgia. His chest x ray indicated severe pneumonia and ground-glass opacities, consistent with COVID-19. On admission, he had a peripheral capillary oxygen saturation (SpO_2_) of 84% with oxygen support. He received 7 liters oxygen/min to keep his SpO_2_ at 78–84%. The patient was initially prescribed hydroxychloroquine and azithromycin. On day 3 of hospitalization, his body temperature increased to 39°C. Favipravir was added to the regimen, followed by tocilizumab. Tocilizumab was administered for 2 d and then stopped. Favipravir was stopped on day 5, and the fever subsided on the third day of treatment. The need for oxygen supplementation eventually subsided, and the patient was monitored, without oxygen, for 15 d after admission. He was discharged from the hospital after 17 d. He has BMI of 27.3 kg/m^2^, does not smoke, and has no comorbidities (no asthma, recurrent infections, immunodeficiency, or hypogammaglobulinemia).

#### Kindred D

Patient P4, an Iranian man born in 1991, presented with flu-like symptoms, including cough, fever, and dyspnea of 7 d duration before admission. PCR on a nasopharyngeal sample was positive for SARS-CoV-2. Upon admission, the patient had a SpO_2_ of 60% and was intubated in the emergency ward. He was admitted to the ICU, where he was placed on mechanical ventilation. He received IFN-β1a (initiated at admission, 24 million IU equivalent, three doses), methylprednisolone (40 mg/d), and hemoperfusion. He died 20 d after admission. He had an unremarkable medical history, a BMI of 29.4 kg/m^2^, did not smoke, and had no comorbid conditions other than being overweight (no asthma, recurrent infections, immunodeficiency, or hypogammaglobulinemia).

#### Kindred E

Patients P5 and P6, two brothers of Swedish/Finnish ethnic origin, born in 1982 and 1989, respectively, suffered from critical/severe SARS-CoV-2 infections during the first wave of the COVID-19 pandemic in Stockholm, Sweden, in the spring of 2020.

The elder brother (P5) was previously healthy, with no significant infection episodes. As a child, he was vaccinated in accordance with the Swedish vaccination program, which includes live MMR vaccines. In March 2020, he developed dyspnea, cough, fever, chest pain, impaired smell and taste, diarrhea, fatigue, and muscle and joint pain. Contact tracing suggested that he was infected by a colleague who had recently visited Austria. 10 d after the onset of symptoms, he was admitted to the infectious disease clinic at a tertiary hospital. A PCR test for SARS-CoV-2 was positive, as subsequently confirmed by a positive serological test for IgG anti-spike receptor-binding domain antibodies. Laboratory tests at admission showed high CRP levels (402 mg/liter), a high leukocyte count (11.6 × 10^9^/liter, including 9.7 × 10^9^ neutrophils/liter), and lymphopenia (0.6 × 10^9^/liter). Chest x ray showed pronounced bilateral pulmonary opacification, predominantly in the lower lobes. Patient P5 suffered respiratory failure and was transferred to the ICU after 2 d. He required intubation with assisted ventilation for 5 d. He spent 15 d in the ICU before returning to the infectious disease clinic. During hospitalization, he received chloroquine phosphate and antibiotics (erythromycin, cefotaxime, ceftriaxone, meropenem), but no concomitant bacterial infections were detected. He was discharged home after 23 d in hospital.

3 wk after discharge, he sought medical assistance for heart palpitations. Transient myocardial damage was suspected; exercise electrocardiogram and troponin levels were normal on follow-up. He suffered from mild dyspnea, mild fatigue, and body aches for a few months but, with the exception of mild fatigue, he had almost fully recovered 6 mo after admission and had returned to work full-time. Laboratory tests performed 7 mo after admission showed normal counts of leukocytes (4.5 × 10^9^/liter), neutrophils (2.6 × 10^9^/liter), and total lymphocytes (1,500 × 10^6^/liter). Immunoglobulin analyses were normal, with 0.97 g/liter IgM, 11.1 g/liter IgG, and 4.1 g/liter IgA. No autoantibodies against IFN-α2 were detected.

The younger brother of patient P5 (P6) had had recurrent ear infections during his early school years, requiring the insertion of a tympanostomy tube. He had also been vaccinated according to the Swedish vaccination program for children. During his teenage years, he suffered from two severe infectious episodes: one influenza episode requiring hospitalization at the age of 14 yr, and one streptococcal infection requiring hospitalization and antibiotic treatment. In the middle of April 2020, he developed dyspnea, fever, muscle pain, and a sore throat. 7 d later, he was admitted to the infectious disease clinic of a tertiary university hospital. A PCR test for SARS-CoV-2 was positive and was subsequently confirmed by a positive serological test for IgG anti-spike receptor-binding domain antibodies. Laboratory tests performed on admission showed high CRP levels (306 mg/liter), a high leukocyte count (11.6 × 10^9^/liter, including 7.2 × 10^9^ neutrophils/liter), and lymphopenia (0.6 × 10^9^/liter). IL-6 levels were 7.7 ng/liter and procalcitonin levels were 0.63 µg/liter. The patient’s body temperature reached 41.5°C. Oxygen treatment was sufficient to maintain adequate blood oxygen saturation and intubation was never needed. Tocilizumab treatment was considered, but never administered, after rapid improvement on day 4 of hospitalization. A chest CT scan showed pronounced bilateral pulmonary opacification that was predominantly peripheral and in the lower lobes, more pronounced on the left side. No pulmonary embolism was detected. The patient received cefotaxime and ceftriaxone, but no concomitant bacterial infections were found. The patient was discharged home after 4 d of hospitalization and was able to return to work after 10 d of sick leave.

8 mo after admission, he continued to feel a slight loss of stamina but had otherwise fully recovered. Laboratory tests results were normal (5.3 × 10^9^/liter leukocytes, 3.7 × 10^9^/liter neutrophils, and 1.300 × 10^9^/liter total lymphocytes). Immunoglobulin analyses showed IgM levels (0.43 g/liter) and IgG levels (9.7 g/liter) to be normal and IgA levels (4.6 g/liter) to be slightly high. No autoantibodies against IFN-α2 were detected.

In the middle of August 2021, patient P6 developed a severe headache with fever. After 4 d, a sudden improvement was observed that lasted 2 d before the symptoms worsened again. 9 d after the onset of the initial symptoms, patient P6 developed diplopia and was admitted to the infectious disease clinic at a tertiary university hospital. He had no signs of respiratory infection or cognitive impairment. During the weeks before he developed symptoms, patient P6 had been on vacation in the Stockholm archipelago, where he had received several tick bites. He had received his third dose of tick-borne encephalitis (TBE) vaccine 1 mo before the onset of symptoms. On admission, he had a CRP concentration of 10 mg/liter, a high leukocyte count (10.8 × 10^9^/liter including 8.3 × 10^9^ neutrophils/liter), and a normal lymphocyte count (1.6 × 10^9^/liter). Cerebrospinal fluid (CSF) analyses revealed pleocytosis, with a high leukocyte count (248 × 10^6^/liter, including 226 × 10^6^ monocytes/liter), and an albumin concentration of 745 mg/liter. Serological tests on blood and CSF (IgG and IgM) were negative for *Borrelia burgdorferi*. Serous meningitis was suspected. PCR for the TBE virus was negative on CSF, but serological tests were positive on both CSF and serum, with high titers of neutralizing antibodies in the serum, suggesting a breakthrough infection despite three doses of vaccine. After 2 d in hospital, the patient’s symptoms improved rapidly; he was discharged home after 3 d and recovered fully.

#### Kindred F

Patient P7 is a Belgian girl born at term in 2016, after an uneventful pregnancy, with a birth weight of 3,570 g and an Apgar score of 9/10. The parents are not known to be consanguineous. At the age of 6 mo, she was admitted to hospital with PCR-proven RSV bronchiolitis, clinically diagnosed on the basis of bilateral wheezing and crackles, complicated by suspected bacterial pneumonia, with bilateral patchy infiltrates on x ray, responding to antibiotic treatment. At the age of 7 mo, patient P7 was hospitalized with IAV bronchopneumonia and a high fever and given oxygen therapy and antibiotic treatment. At the age of 8 mo, she was admitted for sepsis and metabolic acidosis. All cultures (blood, urine, CSF) were negative, and no viral agent was detected in stool samples or throat swabs (limited panel). CRP concentration was high (189.6 g/liter). Empiric treatment with cefotaxim was administered for 6 d in hospital, followed by amoxicillin-clavulinic acid for 5 d. At the age of 21 mo, the patient was treated with amoxicillin-clavulanic acid for otitis media, periorbital cellulitis, and lymphadenitis. At the age of 33 mo, she had scarlet fever (clinical diagnosis), which was treated with oral cefadroxil for 10 d. 1 mo later, she was admitted for high fever, exanthema, and bilateral crackles, corresponding to bilateral bronchopneumonia on x ray. A viral swab revealed adenovirus infection, and a throat swab was positive for *Streptococcus pyogenes*. She was treated with i.v. amoxicillin for 6 d and was discharged on oral amoxicillin. At the age of 3 yr and 1 mo, the patient was again admitted for high fever with cough and dehydration. Chest x ray showed bilateral bronchopneumonia. CRP concentration was 280 mg/liter. The patient was admitted for 1 wk, receiving i.v. amoxicillin-clavulanic acid, and then oral treatment with the same drugs for 5 d. Throat swabs remained adenovirus positive. At the age of 3 yr and 5 mo, the patient received ambulatory treatment with 70 mg/kg amoxicillin for 1 wk for pneumonia (CRP 80 mg/liter). 1 mo later, she underwent a basic immunological assessment elsewhere, which showed normal antibody levels and normal immunophenotyping but weak responses to the unconjugated pneumococcal vaccine. At the age of 3 yr and 9 mo, the patient was again hospitalized, for respiratory distress and high fever, corresponding to yet another episode of bronchopneumonia, with an upper respiratory tract swab testing positive for IAV. At this point, the patient was referred to Leuven University Hospital for immunological assessment. The patient suffered another episode of radiologically proven pneumonia at the age of 46 mo, for which she was treated as an outpatient with amoxicillin/clavulanic acid for 12 d. CRP concentration reached 150 mg/liter.

Upon referral, the patient appeared to be a healthy young girl. Cystic fibrosis and primary ciliary dyskinesia had been excluded as underlying diagnoses. Immune screening was normal except for the borderline anti-pneumococcal response. Laboratory tests showed normal counts of total lymphocytes (3,600 × 10^6^/liter), and lymphocyte profiling detected nothing aberrant. Immunoglobulin analyses showed normal IgM levels of 0.99 g/liter, IgG levels of 9.0 g/liter, and IgA levels of 0.73 g/liter. Trio WES was therefore performed, and the patient was placed on azithromycin prophylaxis while awaiting the results. Annual flu vaccination was recommended, and the patient received two shots of the COVID-19 BNT162b16 vaccine (Pfizer) off-label. Her vaccination schedule is otherwise up to date, and no adverse events were noted following vaccination with MMR. She is currently well and off all treatment. We have discussed the option of immunoglobulin prophylaxis with the parents, but, for now, close clinical observation is preferred.

### Genetics

The methods used for high-throughput sequencing have been described elsewhere (Zhang et al., 2020). In brief, genomic DNA was extracted from whole blood. For WES, libraries were generated with the Twist Bioscience kit (Twist Human Core Exome Kit), the xGen Exome Research Panel from Integrated DNA Technologies (IDT xGen), the Agilent SureSelect V7 kit, or the SeqCap EZ MedExome kit (Roche). Massively parallel sequencing was performed on a NovaSeq6000 system (Illumina). We used the Genome Analysis Software Kit (v3.4-46 or 4; GATK) best-practice pipeline to analyze our WES data (DePristo et al., 2011). We aligned the reads obtained with the human reference genome (hg19), using the maximum exact matches algorithm in the Burrows–Wheeler Aligner (Li and Durbin, 2009). PCR duplicates were removed with Picard tools (http://picard.sourceforge.net). The GATK base quality score recalibrator was applied to correct sequencing artifacts. For P5 and P6, Illumina whole-genome sequencing, mapping, and read processing and annotation were performed according to the molecular inversion probe rare disease pipeline used at the Genomic Medicine Center Karolinska Rare Diseases (Stranneheim et al., 2021). Results were confirmed by Sanger sequencing.

All the variants were manually curated with Integrative Genomics Viewer and confirmed to affect the main functional protein isoform by verifying the protein sequence before inclusion in further analyses. The main functional protein isoform for IRF7 is NM_001572.5 (Zhang et al., 2020).

### Cells

Blood samples were obtained in heparin-treated tubes from patients or from healthy human donor buffy coats or heparin-treated whole-blood samples (Karolinska University Hospital). PBMCs were isolated by density gradient centrifugation (Lymphoprep; STEMCELL Technologies) and resuspended in complete RPMI medium (RPMI 1640 with GlutaMAX [Gibco] supplemented with 10% FBS [Thermo Fisher Scientific]) for further analysis. HEK293T cells were cultured in DMEM supplemented with 10% FBS.

### Evaluation of the stability and function of IRF7 variants in an ectopic expression system

HEK293T cells were cotransfected with a mixture of the IFN-β–firefly luciferase reporter plasmid, the pRL-TK-*Renilla* luciferase plasmid, and the pcDNA3-IRF7 plasmid. Cells were incubated for 24 h and were then either left untreated or infected with Sendai virus (20 units/well) for another 24 h. Reporter activity was measured with the Dual-Luciferase Reporter Assay System, and all experiments were performed in technical triplicates. Firefly luciferase activity was normalized against *Renilla* luciferase activity. IRF7 levels were assessed by Western blotting using anti-IRF7 antibody (clone D2A1J; Cell Signaling) and anti-FLAG antibody (clone M2; Sigma-Aldrich). Membranes were reprobed for HRP-conjugated GAPDH (clone 1E6D9; PTG Lab) as a protein loading control.

### Assessment of IRF7 levels in patients

Thawed PBMCs from cryopreserved samples (1 × 10^6^ cells/well) were stimulated by incubation in complete RPMI in a 24-well plate. Cells were stimulated with 1,000 U/ml IFN-β (PBL Assay Science) or left unstimulated for 24 h at 37°C under an atmosphere containing 5% CO_2_. At the end of the assay, the cells were harvested, washed in PBS, and the cell pellets were frozen at −20°C. Total cell lysates were subsequently prepared by lysis in radioimmunoprecipitation assay buffer (Thermo Fisher Scientific) with the inhibitors PMSF (ChemCruz) and sodium orthovanadate (ChemCruz), on ice for 1 h. The lysates were then denatured by heating at 70°C for 10 min in NuPAGE LDS sample buffer (Invitrogen) with 50 mM 1,4-dithiothreitol (Sigma-Aldrich) and resolved by gel electrophoresis in NuPAGE 4–12% Bis-Tris gels (Thermo Fisher Scientific). The protein bands were then transferred onto polyvinylidene difluoride membranes with the iBlot 2 Dry Blotting System (Thermo Fisher Scientific). Membranes were probed with anti-IRF7 antibody (#4920; Cell Signaling Technology) followed by HRP-conjugated anti-rabbit secondary antibody (Invitrogen). The membranes were then stripped and reprobed with HRP–anti-actin antibody (Invitrogen) as a protein loading control. Bands were visualized by enhanced chemiluminescence with the SuperSignal West Dura Extended Duration Substrate (Thermo Fisher Scientific) on an Odyssey Imaging System (LI-COR Biosciences).

### Antibodies for flow cytometry

We used the following antibodies from BD Biosciences: BUV805–anti-human CD3 (clone UCHT1), BV711–anti-human CD11c (clone B-ly6), BUV395–anti-human CD56 (clone B159), PE-Cy7–anti-human CD95 (clone DX2), and PE–anti-human TNF (clone MAb11). The following antibodies were obtained from BioLegend: BV421–anti-human CCR7 (clone G043H7), BV711–anti-human CD8a (clone SK1), BV510–anti-human CD14 (clone M5E2), BV510–anti-human CD19 (clone HIB19), PE-Cy7–anti-human CD123 (clone 6H6), BV421–anti-human CD303 (BDCA-2; clone 201A), BV785–anti-human HLA-DR (clone L243), and FITC–anti-human IFN-γ (clone 4S.B3). The Qdot605–anti-human CD4 (clone S3.5) antibody was obtained from Thermo Fisher Scientific. The following antibodies were obtained from Miltenyi Biotec: FITC–anti-human CD141 (BDCA-3; clone AD5-14H12) and APC–anti-human IFN-α (clone LT27:295).

### TLR stimulation of DCs

Freshly isolated PBMCs or thawed PBMCs from cryopreserved samples (1.5 × 10^6^ cells/well) were stimulated in complete RPMI in flat-bottom 96-well plates. For cryopreserved cells, stimulations were performed on freshly thawed cells, except for imiquimod stimulations, which were performed after allowing the cells to rest overnight. Cells were stimulated with 4 µg/ml imiquimod (Alfa Aesar), 2 μM CpG ODN 2336 (InvivoGen), 10 μg/ml poly(I:C) (Miltenyi Biotec) or left unstimulated for 6 h in total at 37°C, under an atmosphere containing 5% CO_2_. The addition of GolgiPlug (BD Biosciences) was optimized for each set of conditions. GolgiPlug was added after 1.5 h of stimulation for imiquimod, 3 h after stimulation for poly(I:C), or 4.5 h after stimulation for ODN.

At the end of the assay, cells were collected and incubated with CF430 viability dye (Biotium) and cell-surface antibody cocktails in FACS buffer (PBS supplemented with 1% FBS and 10 mM EDTA) at room temperature for 20 min. Cells were then fixed by incubation in 2% formaldehyde (Sigma-Aldrich) at room temperature for 15 min. The cells were permeabilized by washing twice in BD Perm/Wash Buffer (BD Biosciences) and stained for intracellular markers in Perm/Wash at room temperature for 30 min. Cells were then washed in Perm/Wash and finally resuspended in FACS buffer for acquisition on a BD FACSymphony flow cytometer (BD Biosciences) and analysis with FlowJo software (v10; BD Biosciences).

### Peptide stimulation of T cells

Thawed PBMCs from cryopreserved samples (1 × 10^6^ cells/well) were stimulated in U-bottom 96-well plates with 0.5 μg/ml pooled peptides or 10 ng/ml PMA (Sigma-Aldrich) plus 0.5 μM ionomycin (Sigma-Aldrich) in complete RPMI. The following peptide pools were used: CEFX Ultra SuperStim Pool MHC-I Subset (JPT Peptide Technologies), HCMV IE-1 (JPT Peptide Technologies), HCMV IE-2 (JPT Peptide Technologies), HCMV pp65 (JPT Peptide Technologies), EBV BLZF with 100 μg/ml EBNA1 (Peptides & Elephants), Peptivator H1N1 HA (Miltenyi Biotec), Peptivator H1N1 MP1 (Miltenyi Biotec), Peptivator H1N1 MP2 (Miltenyi Biotec), Peptivator H1N1 NA (Miltenyi Biotec), Peptivator H1N1 NP (Miltenyi Biotec), Peptivator SARS-CoV-2 Prot-N (Miltenyi Biotec), and Peptivator SARS-CoV-2 Prot-S Complete (Miltenyi Biotec). Cells were stimulated for 6 h total at 37°C under an atmosphere containing 5% CO_2_, with the addition of GolgiPlug (BD Biosciences) after 30 min of stimulation. At the end of the assay, cells were collected and incubated with CF430 viability dye (Biotium) and cell-surface antibody cocktail in FACS buffer (PBS supplemented with 1% FBS and 10 mM EDTA) at room temperature for 20 min. Cells were then fixed by incubation in 2% formaldehyde (Sigma-Aldrich) at room temperature for 15 min. The cells were permeabilized by washing twice in BD Perm/Wash Buffer (BD Biosciences), before staining for intracellular markers in Perm/Wash at room temperature for 30 min. The cells were then washed in Perm/Wash and resuspended in FACS buffer for acquisition on a BD FACSymphony flow cytometer (BD Biosciences) and analysis with FlowJo software (v10; BD Biosciences).

### Whole-blood assays of T cell–specific antigen responses

Flow cytometry assays for specific cell-mediated immune responses in activated whole blood were performed as previously described, to quantify CD4^+^ and CD8^+^ T cell blasts after 6 d of antigen stimulation (Lind Enoksson et al., 2021; Marits et al., 2014). Briefly, heparin-treated whole blood was diluted 1:10 in GlutaMAX RPMI 1640 (supplemented with 2 mM L-glutamine, 100 IU/ml penicillin, and 100 IU/ml streptomycin). In a U-bottom 96-well plate, cells were stimulated with influenza inactivated split-virus quadrivalent vaccine (Vaxigrip Tetra; Sanofi Pasteur) or UV-inactivated supernatant from cells infected with SARS-CoV-2 (The Public Health Agency of Sweden) at 37°C under an atmosphere containing 5% CO_2_. After 6 d of incubation, the samples were stained with FITC–anti-human CD3 (clone SK7) and PE–anti-human CD4 (clone SK3) antibodies from BD Simultest (BD Biosciences), and erythrocytes were lysed in IOTest lysing solution (Beckman Coulter). Samples were acquired on a Cytoflex S flow cytometer (Beckman Coulter) and analyzed with KaluzaC software (Beckman Coulter). Blasts were identified as cells with high forward scatter, and the results are expressed as blasts/μl of whole blood.

### VirScan

Patient serum samples were analyzed with VirScan as previously described (Xu et al., 2015). Briefly, an oligonucleotide library encoding 56–amino acid peptides tiling across the genomes of 206 viral species was synthesized on a releasable DNA microarray and cloned in the T7 phage. Patient serum samples containing 2 μg of IgG were added to the phage library, and immunoprecipitation was performed with Protein A and G beads. Peptides displaying enrichment in the samples were identified by PCR and Illumina sequencing of the peptide cassette from the immunoprecipitated phage.

### RNA-seq

Previously published (Zhang et al., 2020; Sequence Read Archive accession no. PRJNA833449) RNA-seq data was reanalyzed to assess the differential expression of type I and type III IFN genes, as well as curated lists of ISGs (van der Wijst et al., 2021) and inflammatory genes (Liberzon et al., 2011), in isolated pDCs from P2 and a healthy control either unstimulated or cultured with SARS-CoV-2 or IAV.

### Statistics

Statistical analyses were performed with Prism software (GraphPad).

### Online supplemental material

Fig. S1 shows RNA-seq analysis of pDCs from P2 and a healthy control cultured with SARS-CoV-2 or IAV, focusing on IFN and inflammatory gene expression. Fig. S2 shows that BDCA-3^+^ DCs from patients P5, P6, and P7 produce levels of TNF similar to healthy controls following PBMC stimulation with TLR3 agonist poly(I:C). Fig. S3 shows patients that P5 and P6 had significantly stronger CD4^+^ T cell blast responses to influenza virus vaccine stimulation than healthy controls and similar T cell blast responses to SARS-CoV-2 compared to healthy convalescent controls. Fig. S3 also depicts VirScan analysis of all patients showing serological evidence of prior exposure to many common pathogens and responses to IAV and coronavirus antigens. Table S1 details the nine biallelic patient-associated *IRF7* variants, all nonsynonymous or splicing site variants of *IRF7* present in a homozygous state in at least one individual reported in the public gnomAD database, and all *IRF7* variants present in a heterozygous state in the gnomAD database with a MAF >0.0001.

## Supplementary Material

Table S1 details the nine biallelic patient-associated IRF7 variants, all nonsynonymous or splicing site variants of IRF7 present in a homozygous state in at least one individual reported in the public gnomAD database, and all IRF7 variants present in a heterozygous state in the gnomAD database with an MAF >0.0001.

SourceData F2 contains original blots for Fig. 2.

SourceData F3 contains original blots for Fig. 3.

## References

[bib1] Abolhassani, H., N. Landegren, P. Bastard, M. Materna, M. Modaresi, L. Du, M. Aranda-Guillen, F. Sardh, F. Zuo, P. Zhang, . 2022. Inherited IFNAR1 deficiency in a child with both critical COVID-19 pneumonia and multisystem inflammatory syndrome. J. Clin. Immunol. 42:471–483. 10.1007/s10875-022-01215-7 35091979 PMC8798309

[bib2] Alosaimi, M.F., M.C. Maciag, C.D. Platt, R.S. Geha, J. Chou, and L.M. Bartnikas. 2019. A novel variant in STAT2 presenting with hemophagocytic lymphohistiocytosis. J. Allergy Clin. Immunol. 144:611–613.e3. 10.1016/j.jaci.2019.05.008 31102697 PMC6688952

[bib3] Asano, T., B. Boisson, F. Onodi, D. Matuozzo, M. Moncada-Velez, M.R.L. Maglorius Renkilaraj, P. Zhang, L. Meertens, A. Bolze, M. Materna, . 2021. X-linked recessive TLR7 deficiency in ∼1% of men under 60 years old with life-threatening COVID-19. Sci. Immunology. 6:eabl4348. 10.1126/sciimmunol.abl4348 34413140 PMC8532080

[bib4] Bastard, P., K.C. Hsiao, Q. Zhang, J. Choin, E. Best, J. Chen, A. Gervais, L. Bizien, M. Materna, C. Harmant, . 2022. A loss-of-function IFNAR1 allele in Polynesia underlies severe viral diseases in homozygotes. J. Exp. Med. 219:e20220028. 10.1084/jem.20220028 35442418 PMC9026234

[bib5] Bastard, P., J. Manry, J. Chen, J. Rosain, Y. Seeleuthner, O. AbuZaitun, L. Lorenzo, T. Khan, M. Hasek, N. Hernandez, . 2021. Herpes simplex encephalitis in a patient with a distinctive form of inherited IFNAR1 deficiency. J. Clin. Invest. 131:e139980. 10.1172/jci139980 PMC7773360 32960813

[bib6] Bidwell, B.N., C.Y. Slaney, N.P. Withana, S. Forster, Y. Cao, S. Loi, D. Andrews, T. Mikeska, N.E. Mangan, S.A. Samarajiwa, . 2012. Silencing of Irf7 pathways in breast cancer cells promotes bone metastasis through immune escape. Nat. Med. 18:1224–1231. 10.1038/nm.2830 22820642

[bib7] Blanco-Melo, D., B.E. Nilsson-Payant, W.C. Liu, S. Uhl, D. Hoagland, R. Moller, T.X. Jordan, K. Oishi, M. Panis, D. Sachs, . 2020. Imbalanced host response to SARS-CoV-2 drives development of COVID-19. Cell. 181:1036–1045.e9. 10.1016/j.cell.2020.04.026 32416070 PMC7227586

[bib8] Boehmer, D.F.R., L.M. Koehler, T. Magg, P. Metzger, M. Rohlfs, J. Ahlfeld, A. Rack-Hoch, K. Reiter, M.H. Albert, S. Endres, . 2020. A novel complete autosomal-recessive STAT1 LOF variant causes immunodeficiency with hemophagocytic lymphohistiocytosis-like hyperinflammation. J. Allergy Clin. Immunol. Pract. 8:3102–3111. 10.1016/j.jaip.2020.06.034 32603902 PMC9188869

[bib9] Bradley, P., and P.G. Thomas. 2019. Using T cell receptor repertoires to understand the principles of adaptive immune recognition. Annu. Rev. Immunol. 37:547–570. 10.1146/annurev-immunol-042718-041757 30699000

[bib10] Burns, C., A. Cheung, Z. Stark, S. Choo, L. Downie, S. White, R. Conyers, and T. Cole. 2016. A novel presentation of homozygous loss-of-function STAT-1 mutation in an infant with hyperinflammation-A case report and review of the literature. J. Allergy Clin. Immunol. Pract. 4:777–779. 10.1016/j.jaip.2016.02.015 27117246

[bib11] Casanova, J.L., and L. Abel. 2021. Mechanisms of viral inflammation and disease in humans. Science. 374:1080–1086. 10.1126/science.abj7965 34822298 PMC8697421

[bib12] Casanova, J.L., H.C. Su, and C.H.G. Effort. 2020. A global effort to define the human genetics of protective immunity to SARS-CoV-2 infection. Cell. 181:1194–1199. 10.1016/j.cell.2020.05.016 32405102 PMC7218368

[bib13] Cella, M., D. Jarrossay, F. Facchetti, O. Alebardi, H. Nakajima, A. Lanzavecchia, and M. Colonna. 1999. Plasmacytoid monocytes migrate to inflamed lymph nodes and produce large amounts of type I interferon. Nat. Med. 5:919–923. 10.1038/11360 10426316

[bib14] Chapgier, A., R.F. Wynn, E. Jouanguy, O. Filipe-Santos, S. Zhang, J. Feinberg, K. Hawkins, J.L. Casanova, and P.D. Arkwright. 2006. Human complete Stat-1 deficiency is associated with defective type I and II IFN responses in vitro but immunity to some low virulence viruses in vivo. J. Immunol. 176:5078–5083. 10.4049/jimmunol.176.8.5078 16585605

[bib15] Chu, H., J.F.-W. Chan, Y. Wang, T.T.-T. Yuen, Y. Chai, Y. Hou, H. Shuai, D. Yang, B. Hu, X. Huang, . 2020. Comparative replication and immune activation profiles of SARS-CoV-2 and SARS-CoV in human lungs: An ex vivo study with implications for the pathogenesis of COVID-19. Clin. Infect. Dis. 71:1400–1409. 10.1093/cid/ciaa410 32270184 PMC7184390

[bib16] Ciancanelli, M.J., L. Abel, S.Y. Zhang, and J.L. Casanova. 2016. Host genetics of severe influenza: From mouse Mx1 to human IRF7. Curr. Opin. Immunol. 38:109–120. 10.1016/j.coi.2015.12.002 26761402 PMC4733643

[bib17] Ciancanelli, M.J., S.X.L. Huang, P. Luthra, H. Garner, Y. Itan, S. Volpi, F.G. Lafaille, C. Trouillet, M. Schmolke, R.A. Albrecht, . 2015. Infectious disease. Life-threatening influenza and impaired interferon amplification in human IRF7 deficiency. Science. 348:448–453. 10.1126/science.aaa1578 25814066 PMC4431581

[bib18] Colonna, M., G. Trinchieri, and Y.J. Liu. 2004. Plasmacytoid dendritic cells in immunity. Nat. Immunol. 5:1219–1226. 10.1038/ni1141 15549123

[bib19] Crouse, J., G. Bedenikovic, M. Wiesel, M. Ibberson, I. Xenarios, D. Von Laer, U. Kalinke, E. Vivier, S. Jonjic, and A. Oxenius. 2014. Type I interferons protect T cells against NK cell attack mediated by the activating receptor NCR1. Immunity. 40:961–973. 10.1016/j.immuni.2014.05.003 24909889

[bib20] Crouse, J., U. Kalinke, and A. Oxenius. 2015. Regulation of antiviral T cell responses by type I interferons. Nat. Rev. Immunol. 15:231–242. 10.1038/nri3806 25790790

[bib21] Duncan, C.J.A., M.K. Skouboe, S. Howarth, A.K. Hollensen, R. Chen, M.L. Borresen, B.J. Thompson, J. Stremenova Spegarova, C.F. Hatton, F.F. Staeger, . 2022. Life-threatening viral disease in a novel form of autosomal recessive IFNAR2 deficiency in the Arctic. J. Exp. Med. 219:e20212427. 10.1084/jem.20212427 35442417 PMC9026249

[bib22] Duncan, C.J.A., S.M.B. Mohamad, D.F. Young, A.J. Skelton, T.R. Leahy, D.C. Munday, K.M. Butler, S. Morfopoulou, J.R. Brown, M. Hubank, . 2015. Human IFNAR2 deficiency: Lessons for antiviral immunity. Sci. Transl. Med. 7:307ra154. 10.1126/scitranslmed.aac4227 PMC4926955 26424569

[bib74] DePristo, M.A., E. Banks, R. Poplin, K.V. Garimella, J.R. Maguire, C. Hartl, A.A. Philippakis, G. del Angel, M.A. Rivas, and M. Hanna. 2011. A framework for variation discovery and genotyping using next-generation DNA sequencing dat. Nat. Genet. 43:491–498. 10.1038/ng.806 21478889 PMC3083463

[bib23] Duncan, C.J.A., R.E. Randall, and S. Hambleton. 2021. Genetic lesions of type I interferon signalling in human antiviral immunity. Trends Genet. 37:46–58. 10.1016/j.tig.2020.08.017 32977999 PMC7508017

[bib24] Dupuis, S., E. Jouanguy, S. Al-Hajjar, C. Fieschi, I.Z. Al-Mohsen, S. Al-Jumaah, K. Yang, A. Chapgier, C. Eidenschenk, P. Eid, . 2003. Impaired response to interferon-alpha/beta and lethal viral disease in human STAT1 deficiency. Nat. Genet. 33:388–391. 10.1038/ng1097 12590259

[bib25] Fung, K.Y., N.E. Mangan, H. Cumming, J.C. Horvat, J.R. Mayall, S.A. Stifter, N. De Weerd, L.C. Roisman, J. Rossjohn, S.A. Robertson, . 2013. Interferon-epsilon protects the female reproductive tract from viral and bacterial infection. Science. 339:1088–1092. 10.1126/science.1233321 23449591 PMC3617553

[bib26] Gao, D., M.J. Ciancanelli, P. Zhang, O. Harschnitz, V. Bondet, M. Hasek, J. Chen, X. Mu, Y. Itan, A. Cobat, . 2021. TLR3 controls constitutive IFN-beta antiviral immunity in human fibroblasts and cortical neurons. J. Clin. Invest. 131:e134529. 10.1172/JCI134529 PMC7773389 33393505

[bib27] Garcia-Sastre, A. 2017. Ten strategies of interferon evasion by viruses. Cell Host Microbe. 22:176–184. 10.1016/j.chom.2017.07.012 28799903 PMC5576560

[bib28] Gothe, F., C.F. Hatton, L. Truong, Z. Klimova, V. Kanderova, M. Fejtkova, A. Grainger, V. Bigley, J. Perthen, D. Mitra, . 2020. A novel case of homozygous (IFNAR1) deficiency with haemophagocytic lymphohistiocytosis. Clin. Infect. Dis. 74:136–139. 10.1093/cid/ciaa1790 PMC8752251 33252644

[bib29] Hambleton, S., S. Goodbourn, D.F. Young, P. Dickinson, S.M.B. Mohamad, M. Valappil, N. McGovern, A.J. Cant, S.J. Hackett, P. Ghazal, . 2013. STAT2 deficiency and susceptibility to viral illness in humans. Proc. Natl. Acad. Sci. USA. 110:3053–3058. 10.1073/pnas.1220098110 23391734 PMC3581986

[bib30] Harris, B.D., J. Schreiter, M. Chevrier, J.L. Jordan, and M.R. Walter. 2018. Human interferon- and interferon-kappa exhibit low potency and low affinity for cell-surface IFNAR and the poxvirus antagonist B18R. J. Biol. Chem. 293:16057–16068. 10.1074/jbc.RA118.003617 30171073 PMC6187621

[bib31] Hatesuer, B., H.T.T. Hoang, P. Riese, S. Trittel, I. Gerhauser, H. Elbahesh, R. Geffers, E. Wilk, and K. Schughart. 2017. Deletion of Irf3 and Irf7 genes in mice results in altered interferon pathway activation and granulocyte-dominated inflammatory responses to influenza a infection. J. Innate Immun. 9:145–161. 10.1159/000450705 27811478 PMC6738875

[bib32] Hernandez, N., G. Bucciol, L. Moens, J. Le Pen, M. Shahrooei, E. Goudouris, A. Shirkani, M. Changi-Ashtiani, H. Rokni-Zadeh, E.H. Sayar, . 2019. Inherited IFNAR1 deficiency in otherwise healthy patients with adverse reaction to measles and yellow fever live vaccines. J. Exp. Med. 216:2057–2070. 10.1084/jem.20182295 31270247 PMC6719432

[bib33] Hernandez, N., I. Melki, H. Jing, T. Habib, S.S.Y. Huang, J. Danielson, T. Kula, S. Drutman, S. Belkaya, V. Rattina, . 2018. Life-threatening influenza pneumonitis in a child with inherited IRF9 deficiency. J. Exp. Med. 215:2567–2585. 10.1084/jem.20180628 30143481 PMC6170168

[bib34] Hoffmann, H.H., W.M. Schneider, and C.M. Rice. 2015. Interferons and viruses: An evolutionary arms race of molecular interactions. Trends Immunol. 36:124–138. 10.1016/j.it.2015.01.004 25704559 PMC4384471

[bib35] Honda, K., and T. Taniguchi. 2006. IRFs: Master regulators of signalling by Toll-like receptors and cytosolic pattern-recognition receptors. Nat. Rev. Immunol. 6:644–658. 10.1038/nri1900 16932750

[bib36] Honda, K., H. Yanai, H. Negishi, M. Asagiri, M. Sato, T. Mizutani, N. Shimada, Y. Ohba, A. Takaoka, N. Yoshida, and T. Taniguchi. 2005. IRF-7 is the master regulator of type-I interferon-dependent immune responses. Nature. 434:772–777. 10.1038/nature03464 15800576

[bib37] Hong, X.X., and G.G. Carmichael. 2013. Innate immunity in pluripotent human cells: Attenuated response to interferon-β. J. Biol. Chem. 288:16196–16205. 10.1074/jbc.M112.435461 23599426 PMC3668775

[bib38] Itan, Y., L. Shang, B. Boisson, M.J. Ciancanelli, J.G. Markle, R. Martinez-Barricarte, E. Scott, I. Shah, P.D. Stenson, J. Gleeson, . 2016. The mutation significance cutoff: Gene-level thresholds for variant predictions. Nat. Methods. 13:109–110. 10.1038/nmeth.3739 26820543 PMC4980758

[bib39] Jongbloed, S.L., A.J. Kassianos, K.J. McDonald, G.J. Clark, X. Ju, C.E. Angel, C.J.J. Chen, P.R. Dunbar, R.B. Wadley, V. Jeet, . 2010. Human CD141+ (BDCA-3)+ dendritic cells (DCs) represent a unique myeloid DC subset that cross-presents necrotic cell antigens. J. Exp. Med. 207:1247–1260. 10.1084/jem.20092140 20479116 PMC2882828

[bib40] Karczewski, K.J., L.C. Francioli, G. Tiao, B.B. Cummings, J. Alfoldi, Q. Wang, R.L. Collins, K.M. Laricchia, A. Ganna, D.P. Birnbaum, . 2020. The mutational constraint spectrum quantified from variation in 141,456 humans. Nature. 581:434–443. 10.1038/s41586-020-2308-7 32461654 PMC7334197

[bib41] Kircher, M., D.M. Witten, P. Jain, B.J. O’Roak, G.M. Cooper, and J. Shendure. 2014. A general framework for estimating the relative pathogenicity of human genetic variants. Nat. Genet. 46:310–315. 10.1038/ng.2892 24487276 PMC3992975

[bib42] LaFleur, D.W., B. Nardelli, T. Tsareva, D. Mather, P. Feng, M. Semenuk, K. Taylor, M. Buergin, D. Chinchilla, V. Roshke, . 2001. Interferon-kappa, a novel type I interferon expressed in human keratinocytes. J. Biol. Chem. 276:39765–39771. 10.1074/jbc.M102502200 11514542

[bib43] Lazear, H.M., J.W. Schoggins, and M.S. Diamond. 2019. Shared and distinct functions of type I and type III interferons. Immunity. 50:907–923. 10.1016/j.immuni.2019.03.025 30995506 PMC6839410

[bib44] Le Voyer, T., S. Sakata, M. Tsumura, T. Khan, A. Esteve-Sole, B.K. Al-Saud, H.E. Gungor, P. Taur, V. Jeanne-Julien, M. Christiansen, . 2021. Genetic, immunological, and clinical features of 32 patients with autosomal recessive STAT1 deficiency. J. Immunol. 207:133–152. 10.4049/jimmunol.2001451 34183371 PMC8702442

[bib45] Li, H., and R. Durbin. 2009. Fast and accurate short read alignment with Burrows-Wheeler transform. Bioinformatics. 25:1754–1760. 10.1093/bioinformatics/btp324 19451168 PMC2705234

[bib46] Liberzon, A., A. Subramanian, R. Pinchback, H. Thorvaldsdottir, P. Tamayo, and J.P. Mesirov. 2011. Molecular signatures database (MSigDB) 3.0. Bioinformatics. 27:1739–1740. 10.1093/bioinformatics/btr260 21546393 PMC3106198

[bib47] Lim, H.K., S.X.L. Huang, J. Chen, G. Kerner, O. Gilliaux, P. Bastard, K. Dobbs, N. Hernandez, N. Goudin, M.L. Hasek, . 2019. Severe influenza pneumonitis in children with inherited TLR3 deficiency. J. Exp. Med. 216:2038–2056. 10.1084/jem.20181621 31217193 PMC6719423

[bib48] Lind Enoksson, S., P. Bergman, J. Klingstrom, F. Bostrom, R. Da Silva Rodrigues, M.E. Winerdal, and P. Marits. 2021. A flow cytometry-based proliferation assay for clinical evaluation of T-cell memory against SARS-CoV-2. J. Immunol. Methods. 499:113159. 10.1016/j.jim.2021.113159 34597619 PMC8484816

[bib49] Liu, Y.J. 2001. Dendritic cell subsets and lineages, and their functions in innate and adaptive immunity. Cell. 106:259–262. 10.1016/s0092-8674(01)00456-1 11509173

[bib50] Marits, P., A.C. Wikstrom, D. Popadic, O. Winqvist, and S. Thunberg. 2014. Evaluation of T and B lymphocyte function in clinical practice using a flow cytometry based proliferation assay. Clin. Immunol. 153:332–342. 10.1016/j.clim.2014.05.010 24909732

[bib51] Meyts, I., and J.L. Casanova. 2021. Viral infections in humans and mice with genetic deficiencies of the type I IFN response pathway. Eur. J. Immunol. 51:1039–1061. 10.1002/eji.202048793 33729549 PMC8900014

[bib52] Miyamoto, M., T. Fujita, Y. Kimura, M. Maruyama, H. Harada, Y. Sudo, T. Miyata, and T. Taniguchi. 1988. Regulated expression of a gene encoding a nuclear factor, IRF-1, that specifically binds to IFN-beta gene regulatory elements. Cell. 54:903–913. 10.1016/s0092-8674(88)91307-4 3409321

[bib53] Moens, L., L. Van Eyck, D. Jochmans, T. Mitera, G. Frans, X. Bossuyt, P. Matthys, J. Neyts, M. Ciancanelli, S.Y. Zhang, . 2017. A novel kindred with inherited STAT2 deficiency and severe viral illness. J. Allergy Clin. Immunol. 139:1995–1997.e9. 10.1016/j.jaci.2016.10.033 28087227

[bib54] Poyhonen, L., J. Bustamante, J.L. Casanova, E. Jouanguy, and Q. Zhang. 2019. Life-threatening infections due to live-attenuated vaccines: Early manifestations of inborn errors of immunity. J. Clin. Immunol. 39:376–390. 10.1007/s10875-019-00642-3 31123910 PMC7192346

[bib55] Rapaport, F., B. Boisson, A. Gregor, V. Beziat, S. Boisson-Dupuis, J. Bustamante, E. Jouanguy, A. Puel, J. Rosain, Q. Zhang, . 2021. Negative selection on human genes underlying inborn errors depends on disease outcome and both the mode and mechanism of inheritance. Proc. Natl. Acad. Sci. USA. 118:e2001248118. 10.1073/pnas.2001248118 33408250 PMC7826345

[bib56] Reizis, B. 2019. Plasmacytoid dendritic cells: Development, regulation, and function. Immunity. 50:37–50. 10.1016/j.immuni.2018.12.027 30650380 PMC6342491

[bib57] Sakata, S., M. Tsumura, T. Matsubayashi, S. Karakawa, S. Kimura, M. Tamaura, T. Okano, T. Naruto, Y. Mizoguchi, R. Kagawa, . 2020. Autosomal recessive complete STAT1 deficiency caused by compound heterozygous intronic mutations. Int. Immunol. 32:663–671. 10.1093/intimm/dxaa043 32603428

[bib58] Schneider, W.M., M.D. Chevillotte, and C.M. Rice. 2014. Interferon-stimulated genes: A complex web of host defenses. Annu. Rev. Immunol. 32:513–545. 10.1146/annurev-immunol-032713-120231 24555472 PMC4313732

[bib59] Schoggins, J.W. 2019. Interferon-stimulated genes: What do they all do? Annu. Rev. Virol. 6:567–584. 10.1146/annurev-virology-092818-015756 31283436

[bib60] Shingai, M., T. Ebihara, N.A. Begum, A. Kato, T. Honma, K. Matsumoto, H. Saito, H. Ogura, M. Matsumoto, and T. Seya. 2007. Differential type I IFN-inducing abilities of wild-type versus vaccine strains of measles virus. J. Immunol. 179:6123–6133. 10.4049/jimmunol.179.9.6123 17947687

[bib61] Siegal, F.P., N. Kadowaki, M. Shodell, P.A. Fitzgerald-Bocarsly, K. Shah, S. Ho, S. Antonenko, and Y.J. Liu. 1999. The nature of the principal type 1 interferon-producing cells in human blood. Science. 284:1835–1837. 10.1126/science.284.5421.1835 10364556

[bib62] Sontag, S., M. Forster, J. Qin, P. Wanek, S. Mitzka, H.M. Schuler, S. Koschmieder, S. Rose-John, K. Sere, and M. Zenke. 2017. Modelling IRF8 deficient human hematopoiesis and dendritic cell development with engineered iPS cells. Stem Cell. 35:898–908. 10.1002/stem.2565 28090699

[bib63] Stranneheim, H., K. Lagerstedt-Robinson, M. Magnusson, M. Kvarnung, D. Nilsson, N. Lesko, M. Engvall, B.M. Anderlid, H. Arnell, C.B. Johansson, . 2021. Integration of whole genome sequencing into a healthcare setting: High diagnostic rates across multiple clinical entities in 3219 rare disease patients. Genome Med. 13:40. 10.1186/s13073-021-00855-5 33726816 PMC7968334

[bib64] Vairo, D., L. Tassone, G. Tabellini, N. Tamassia, S. Gasperini, F. Bazzoni, A. Plebani, F. Porta, L.D. Notarangelo, S. Parolini, . 2011. Severe impairment of IFN-gamma and IFN-alpha responses in cells of a patient with a novel STAT1 splicing mutation. Blood. 118:1806–1817. 10.1182/blood-2011-01-330571 21772053

[bib65] van der Wijst, M.G.P., S.E. Vazquez, G.C. Hartoularos, P. Bastard, T. Grant, R. Bueno, D.S. Lee, J.R. Greenland, Y. Sun, R. Perez, . 2021. Type I interferon autoantibodies are associated with systemic immune alterations in patients with COVID-19. Sci. Transl. Med. 13:eabh2624. 10.1126/scitranslmed.abh2624 34429372 PMC8601717

[bib66] Weekes, M.P., P. Tomasec, E.L. Huttlin, C.A. Fielding, D. Nusinow, R.J. Stanton, E.C.Y. Wang, R. Aicheler, I. Murrell, G.W.G. Wilkinson, . 2014. Quantitative temporal viromics: An approach to investigate host-pathogen interaction. Cell. 157:1460–1472. 10.1016/j.cell.2014.04.028 24906157 PMC4048463

[bib67] Wu, X., V.L. Dao Thi, Y. Huang, E. Billerbeck, D. Saha, H.H. Hoffmann, Y. Wang, L.A.V. Silva, S. Sarbanes, T. Sun, . 2018. Intrinsic immunity shapes viral resistance of stem cells. Cell. 172:423–438.e25. 10.1016/j.cell.2017.11.018 29249360 PMC5786493

[bib68] Xu, G.J., T. Kula, Q. Xu, M.Z. Li, S.D. Vernon, T. Ndung’u, K. Ruxrungtham, J. Sanchez, C. Brander, R.T. Chung, . 2015. Viral immunology. Comprehensive serological profiling of human populations using a synthetic human virome. Science. 348:aaa0698. 10.1126/science.aaa0698 26045439 PMC4844011

[bib69] Zhang, P., B. Bigio, F. Rapaport, S.Y. Zhang, J.L. Casanova, L. Abel, B. Boisson, and Y. Itan. 2018. PopViz: A webserver for visualizing minor allele frequencies and damage prediction scores of human genetic variations. Bioinformatics. 34:4307–4309. 10.1093/bioinformatics/bty536 30535305 PMC6289133

[bib70] Zhang, Q., P. Bastard, C.H.G. Effort, A. Cobat, and J.L. Casanova. 2022. Human genetic and immunological determinants of critical COVID-19 pneumonia. Nature. 603:587–598. 10.1038/s41586-022-04447-0 35090163 PMC8957595

[bib71] Zhang, Q., P. Bastard, Z. Liu, J. Le Pen, M. Moncada-Velez, J. Chen, M. Ogishi, I.K.D. Sabli, S. Hodeib, C. Korol, . 2020. Inborn errors of type I IFN immunity in patients with life-threatening COVID-19. Science. 370:eabd4570. 10.1126/science.abd4570 32972995 PMC7857407

[bib72] Zhang, S.Y., E. Jouanguy, S. Ugolini, A. Smahi, G. Elain, P. Romero, D. Segal, V. Sancho-Shimizu, L. Lorenzo, A. Puel, . 2007. TLR3 deficiency in patients with herpes simplex encephalitis. Science. 317:1522–1527. 10.1126/science.1139522 17872438

[bib73] Zhao, Y., W. Chen, W. Zhu, H. Meng, J. Chen, and J. Zhang. 2017. Overexpression of interferon regulatory factor 7 (IRF7) reduces bone metastasis of prostate cancer cells in mice. Oncol. Res. 25:511–522. 10.3727/096504016X14756226781802 27733217 PMC7841009

