# Correction: Respiratory viral infections in otherwise healthy humans with inherited IRF7 deficiency

**DOI:** 10.1084/jem.2022020210282022c

**Published:** 2022-11-07

**Authors:** Tessa Mollie Campbell, Zhiyong Liu, Qian Zhang, Marcela Moncada-Velez, Laura E. Covill, Peng Zhang, Ilad Alavi Darazam, Paul Bastard, Lucy Bizien, Giorgia Bucciol, Sara Lind Enoksson, Emmanuelle Jouanguy, Şemsi Nur Karabela, Taushif Khan, Yasemin Kendir-Demirkol, Andres Augusto Arias, Davood Mansouri, Per Marits, Nico Marr, Isabelle Migeotte, Leen Moens, Tayfun Ozcelik, Isabelle Pellier, Anton Sendel, Sevtap Şenoğlu, Mohammad Shahrooei, C.I. Edvard Smith, Isabelle Vandernoot, Karen Willekens, Kadriye Kart Yaşar, COVID Human Genetic Effort, Peter Bergman, Laurent Abel, Aurélie Cobat, Jean-Laurent Casanova, Isabelle Meyts, Yenan T. Bryceson

Vol. 219, No. 7 | https://doi.org/10.1084/jem.20220202 | June 7, 2022

The authors regret that Sevtap Şenoğlu and Kadriye Kart Yaşar were inadvertently omitted from the author list in the original version of their article. The corrected author list appears above.

The error appears in print and in PDFs downloaded before October 28, 2022.

