# Supplementary material for: Respiratory viral infections in otherwise healthy humans with inherited IRF7 deficiency
Source: J Exp Med. 2022 Jun 7;219(7):e20220202. doi: 10.1084/jem.20220202 (PMC9178406; doi:10.1084/jem.20220202)
Supplement: SourceData F2 — contains original blots for Fig. 2. [file JEM_20220202_SourceDataF2.pdf]

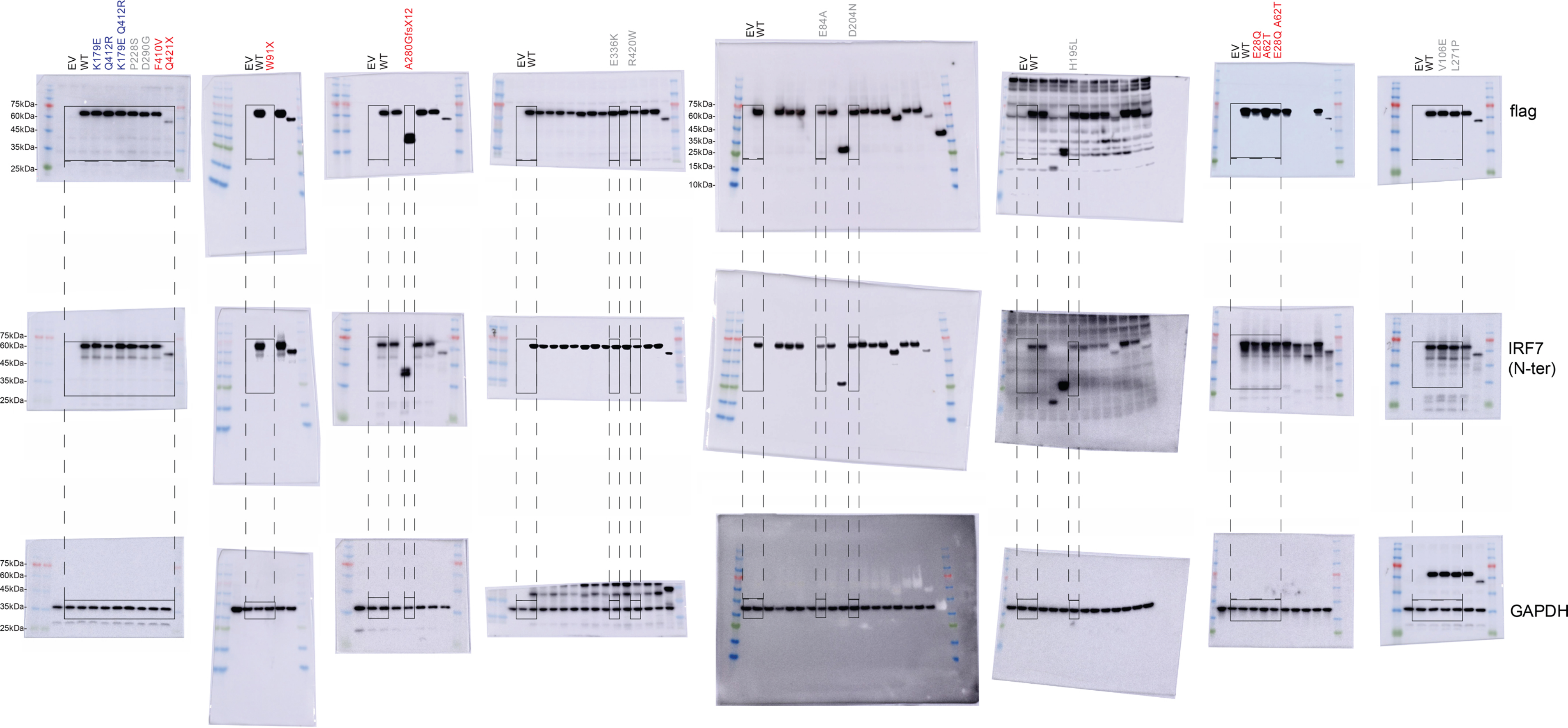

Source Data F2. Campbell, Liu, Zhang *et al.*
